# Individual and collective learning in groups facing danger

**DOI:** 10.1038/s41598-022-10255-3

**Published:** 2022-04-13

**Authors:** Hirokazu Shirado

**Affiliations:** School of Computer Science, Carnegie Mellon University, 5000 Forbes Ave, Newell-Simon Hall 3607, Pittsburgh, PA 15213 USA

**Keywords:** Psychology and behaviour, Evolutionary ecology, Dynamic networks, Human behaviour

## Abstract

While social networks jeopardize people’s well-being by working as diffusion pathways of falsehood, they may also help people overcome the challenge of misinformation with time and experience. Here I examine how social networks provide learning facilitation using an experiment involving an iterated decision-making game simulating an unpredictable situation faced by a group (2786 subjects in 120 groups). This study shows that, while social networks initially spread false information and suppress necessary actions, with tie rewiring, on the other hand, they facilitate improvement in people's decision-making across time. It also shows that the network's learning facilitation results from the integration of individual experiences into structural changes. In sum, social networks can support collective learning when they are built through people's experiences and accumulated relationships.

## Introduction

Social networks spread information and behavior, whether it is good or bad. While people benefit from a social network that passes on valuable information^1^, health practices^2^, and social support^3^, they also suffer because of the spread of fake news^4,5^, ill-health^6^, and violence^7^. Social networks, working as diffusion pathways, can pose a grave challenge to collective decision-making^8^.

In addition to serving as diffusion pathways, however, social networks can play another role in our sociality: collective learning^9^. Theory suggests that social networks can capture collective intelligence *through repeated experience*. People can change their perceptions and behavior not only from their own experience, but also from observations about others^10^. They can also put different weights and trust in different people—and choose to connect or disconnect with them—as they get to know each other^11,12^. For instance, tie rewiring helps people overcome social dilemmas to develop cooperation^13–16^. Social networks also can facilitate other benevolent collective behavior, such as trust, reciprocity^17^, and moral behavior^18^.

This capability of collective learning can be vital for humans (and other animals) to survive in hazardous environments^10,19,20^. In such an environment, collective dangers can occur in succession; e.g., earthquakes that cause tsunamis^21^; or repeated epidemics of disease^22^. When facing one crisis after another, people could increase their fitness by co-evolving their information network and behavior. How might social networks help people learn from past failures in responding to dangers and make better decisions in the future?

Here, I test the learning facilitation of social networks using a laboratory experiment with human subjects. In the experiment, I use adverse conditions that make precise communications and rational decisions difficult in the face of “disaster” risk^23^. In emergencies, people often procrastinate rather than promptly follow necessary evacuation orders, and they promulgate false reassurance, known as normalcy bias^24,25^. Prior work shows that social networks can amplify the harmful bias via interpersonal communication^26^. On the other hand, such a crisis leads people to re-shape their networks^27–29^. Thus, a decision-making scenario simulating a sudden disaster is suitable for the study of collective learning to examine how people overcome the negative influences of social connections.

Social networks can facilitate collective intelligence by two nonexclusive mechanisms^12,19^: (i) a change in internal rules or probabilities of response to neighbors’ signals (i.e., individual learning); (ii) a change in population structure that enhances signal diffusion (i.e., network evolution). The mechanisms correspond to the elements of a graph, i.e., vertices and edges, respectively. Although network theory often evaluates structure separate from individuals, the integration of individuals and network structure, rather than the individuals alone or the network structure alone, might be a key consideration for collective learning^30^. Thus, this study investigates the interaction effect between individuals and network structure by controlling each mechanism with experimental manipulations.

As a causal pathway, each individual can improve their performance as they learn from past experience^19^. In that case, their learning performance should depend on environmental and historical contexts that shape personal experiences^28,29^. Empirical work shows that people respond to a disaster differently depending on prior experience^31,32^. Here, I examine individual responsiveness by manipulating the temporal pattern of “disasters” that subjects have experienced.

Network structure also can affect individual and group judgment in emergencies^33,34^. Prior work suggests that networks with more connections, shorter path lengths, and more clusters can facilitate collective performance^35–37^. Social interactions also can improve the accuracy of group estimates when specific individuals making more accurate estimates have more connections and thus are more influential than others^38^. Network dynamics might thus help people to modify their local connections to develop such an effective network with appropriate feedback^12,27^. Here, we also test these theoretical predictions regarding the impact of network structure on the ability of a group to share accurate information.

### Experiment setup

I recruited 2786 unique subjects via an online labor market (see Supplementary Table 1 for the demographics) and placed them in 120 groups with an average size of 15.5 (s.d. = 1.1). All the subjects consented and passed a series of human verification checks and a screening test of understanding game rules and payoffs before playing the game. They could participate in the experiment only once (see “Methods” for the details).

**Figure 1:**
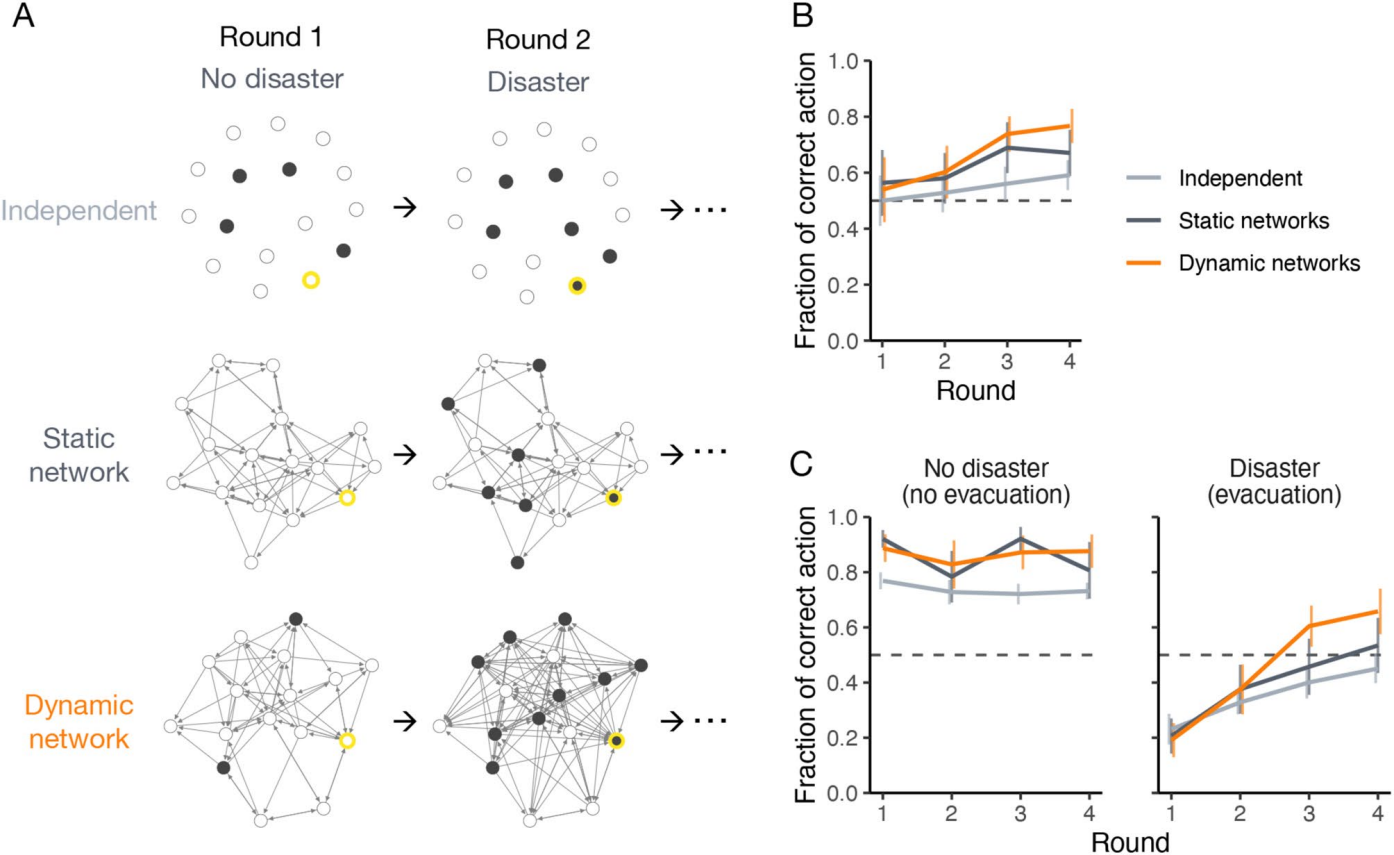
Network condition and collective performance across rounds. (**A**) The states of the first two rounds in three out of 180 sessions. Open nodes indicate players staying to the round’s end; filled ones indicate players evacuating before it. Yellow outline frame indicates informants (who know the truth in advance). Players could update their connections between rounds in the dynamic network condition. (**B**) Overall behavioral accuracy across rounds. Lines indicate average fractions of subjects who select an appropriate behavior at the game end (i.e., stay without “disaster” or evacuate with “disaster”) across rounds with and without a network (*n* = 40 for each condition). The dashed line is 50% of accuracy that indicates the same level as a random binary choice. Error bars are 95% confidence intervals among sessions. (**C**) The changes in behavioral accuracy are highlighted by the presence or absence of “disaster” (*n* = 20 for each condition).

To study collective learning within networks, I used an iterated decision-making game initially developed as a one-shot game to study collective communication and behavior in response to uncertain “disaster,” called the *evacuation game*^23^. In contrast to the standard risky-choice task used to study human decision-making with risk^39,40^, the game captures various dilemmas that people face in real emergencies under time-critical constraints. For instance, people need to decide whether to take costly action (e.g., evacuation) with incomplete information at some point to avoid possible future loss^25^; taking time to collect more information can increase the risk of being adversely affected^41^; and helping others (e.g., staying on-site to communicate newsworthy information) can decrease their own chance of survival^42^. Here, subjects played an iterated evacuation game lasting four rounds in their assigned group. I then manipulated two key dimensions: (i) the temporal pattern of “disasters” (environmental contexts) and (ii) whether and how a group of people had networks for interpersonal communication (group settings) (Fig. 1A).

In each round, subjects received US$1.00 at the outset. If nothing happened until the game suddenly ended, they kept the endowment. However, they might be involved in a “disaster” that could strike at any second. Each subject could spend US$0.50 to avoid this danger at any time. In addition, when subjects were not involved in a “disaster,” they earned US$0.05 for every *other* player who took correct action (Supplementary Table 2). This additional payment incentivized the communication of reliable information about the “disaster” to help others. Each round ended at 75.0 s on average (s.d. = 9.5) without prior notice. After a round ends, subjects knew whether a “disaster” stroke and whether they took correct action (i.e., staying to the end without a “disaster” or evacuating before a “disaster” strikes). Then, subjects played another round until they completed four total rounds.

Within this basic setup, I manipulated the frequency and continuity of “disasters.” A group of subjects experienced possible “disasters” across rounds in one of the following arrangements: a “disaster” materialized every round; a “disaster” did not materialize every round; a “disaster” materialized at round 1 and round 3 and did not metalize at round 2 and round 4; and, finally, a “disaster” materialized at round 2 and round 4 and did not metalize at round 1 and round 3. As a result, a “disaster” struck in precisely half the rounds (i.e., with the probability of 50% for each round). Subjects perceived the “disasters” at random, although the pattern was predetermined (except for randomly selected informants; see below).

Independent of the foregoing disaster patterns, I also manipulated the existence of network connections in a group. I randomly assigned a subject group to one of the three conditions: *independent*, *static network*, or *dynamic network* (Fig. 1A). While the independent condition afforded no inter-personal communication, the network conditions allowed subjects to communicate with each other. In all conditions, at the start of the game, I randomly selected one subject per group to tell the subject (the “informant”) in advance whether a “disaster” would indeed strike. The other subjects did not know who the informant was, but they knew that some players indeed had such accurate information. The group had the same informant across the four rounds (except for a supplement condition; see below). In the independent condition, however, subjects had no network connections to share the accurate information originally from the informant and needed to make an evacuation decision alone.

On the other hand, in the network conditions (static and dynamic networks), the informant, working with others, could pass on the truth about the impending calamity through network connections. Subjects played the game in a *directed* network with a random graph configuration as the initial density was set to 0.25. During the game, they were allowed to share information about the possibly impending “disaster” with neighbors by using “Safe” and “Danger” buttons that indicated their assessment (see “Methods”). Subjects could use the Safe and Danger buttons any time unless they evacuated or did not have to.

I tested two types of network conditions (Fig. 1A). In the *static* network condition, subjects played the four rounds with the same network neighbors. In the *dynamic* network condition, subjects could change their neighbors based on their performance in the last round. After each round, I randomly chose 40% of all pairs of subjects (in both directions). Subjects could rewire the selected connections^14^. If a tie already existed, the predecessor subject (i.e., “follower”) was allowed to choose whether to break the tie with the successor (i.e., “followee”). If a tie did not exist, the predecessor got an option to make a tie with the successor. When making this decision, subjects were informed of the last round’s performance of the focal players (see “Methods”).

To clarify mechanisms for dynamic networks to facilitate collective intelligence, I added two additional conditions to the experiment. In one condition, subjects played the game in the same settings as the dynamic network condition except that informants (i.e., subjects informed about “disasters” in advance) changed every round. I conducted 20 sessions in the random-informant condition (316 subjects in 20 groups; 5 groups each for the four types of “disaster” patterns). In the other condition, subjects were assigned to one of the 40 isomorphic networks that other subjects had developed with tie-rewiring options through the three rounds in the dynamic network condition (567 subjects in 40 groups). Network structure and other game settings (i.e., whether a disaster stroke, how long the game was, and which node was the informant) were identical to where the others played the game at the final round. However, players were different, and they had no prior experience in the game. They simply played the game in a network with a topology created by others ostensibly to optimize the accurate flow of information. In contrast to other conditions, subjects played only one round in the isomorphic network condition.

In sum, I evaluated 14 conditions. The main study had 12 treatment combinations of environmental context and group setting: 4 temporal patterns of “disasters” crossed with three types of group settings (Fig. 1A). I conducted 10 sessions for each treatment combination (i.e., 120 sessions in total). I also conducted one additional condition with random informants (20 sessions) and the other with isomorphic networks (40 sessions). The experiment had a total of 180 sessions with 2786 subjects. Each subject played only one session consisting of four rounds of the evacuation game.

## Results

### Changes in evacuation decisions with networks

Dynamic social networks significantly improved collective performance under risky and uncertain conditions in the experiment (Fig. 1B). In all the conditions, the choice accuracy increased across rounds from about 50% in the first round. However, the improvement with dynamic networks was significantly better than that of the sessions lacking network connections and related communication. The sessions of static networks had marginally significant improvement, compared to the independent sessions (the fraction of correct action in Round 4 = 0.591 ± 0.053 with 95% CI in the independent condition with *n* = 40; 0.671 ± 0.083 in the static network condition; 0.767 ± 0.061 in the dynamic network condition; *n* = 40 for each condition; *P* = 0.028 in the static network condition; *P* < 0.001 in the dynamic network condition; generalized linear mixed model (GLMM) with *n* = 120; see Supplementary Table 3).

The difference between independent and collective learning is highlighted when we examine the evacuation decisions depending on the presence or absence of “disaster” (Fig. 1C). When a “disaster” would not strike, most subjects successfully did not evacuate at the first round, especially in a network (the fraction of correct stay in Round 1= 0.921 ± 0.032 with 95% CI in the static network condition and 0.887 ± 0.050 in the dynamic network condition, compared to 0.769 ± 0.031 in the independent condition; *P* < 0.001 for both comparisons with Welch two-sample *t* test; *n* = 20 for each condition). By contrast, when a “disaster” would indeed strike, about 80% of subjects failed to evacuate at the first round (the fraction of correct evacuation in Round 1 = 0.206 ± 0.064 with 95% CI in the static network condition and 0.191 ± 0.062 in the dynamic network condition, compared to 0.231 ± 0.056 in the independent condition; *P* = 0.563 and *P* = 0.349 for each comparison with Welch two-sample *t* test; *n* = 20 for each condition). These findings are consistent with prior work^23^: interpersonal communication reduces unnecessary evacuations when there is no danger, but it does not promote necessary evacuations when there is indeed a danger, even though an informant, working with others, could pass on the true warnings.

However, social networks with tie rewiring exerted a positive impact on necessary evacuations after subjects repeatedly played the game (Fig. 1C). When a “disaster” would strike, subjects increased the proportion of evacuation through the four rounds by 0.220 ± 0.087 with 95% CI in the independent condition, by 0.328 ± 0.126 in the static network condition, and by 0.467 ± 0.119 in the dynamic network condition (*P* < 0.001 in all conditions with *n* = 20; paired *t* test). While the improvement in evacuation rate was significantly larger in the dynamic network condition than in the independent condition (*P* = 0.001; Welch two-sample *t* test), there was no statistically significant difference between the independent and static network conditions (*P* = 0.146; Welch two-sample *t* test).

On the other hand, when a “disaster” would not strike, there were no meaningful changes in the evacuation rate across rounds in all the conditions (*P* = 0.157 in the independent condition; *P* = 0.061 in the static network condition; *P* = 0.763 in the dynamic network condition; paired *t* test with *n* = 20). More than 80% players always made the same decision without “disaster” (83.6% in the independent condition; 82.5% in the static network condition; 87.0% in the dynamic network condition). I confirmed the consistency of results with a survival analysis examining entire game periods (Supplementary Fig. 1) and a comprehensive analysis using GLMM (Supplementary Table 3).

### Changes in signal diffusion with “disasters”

**Figure 2:**
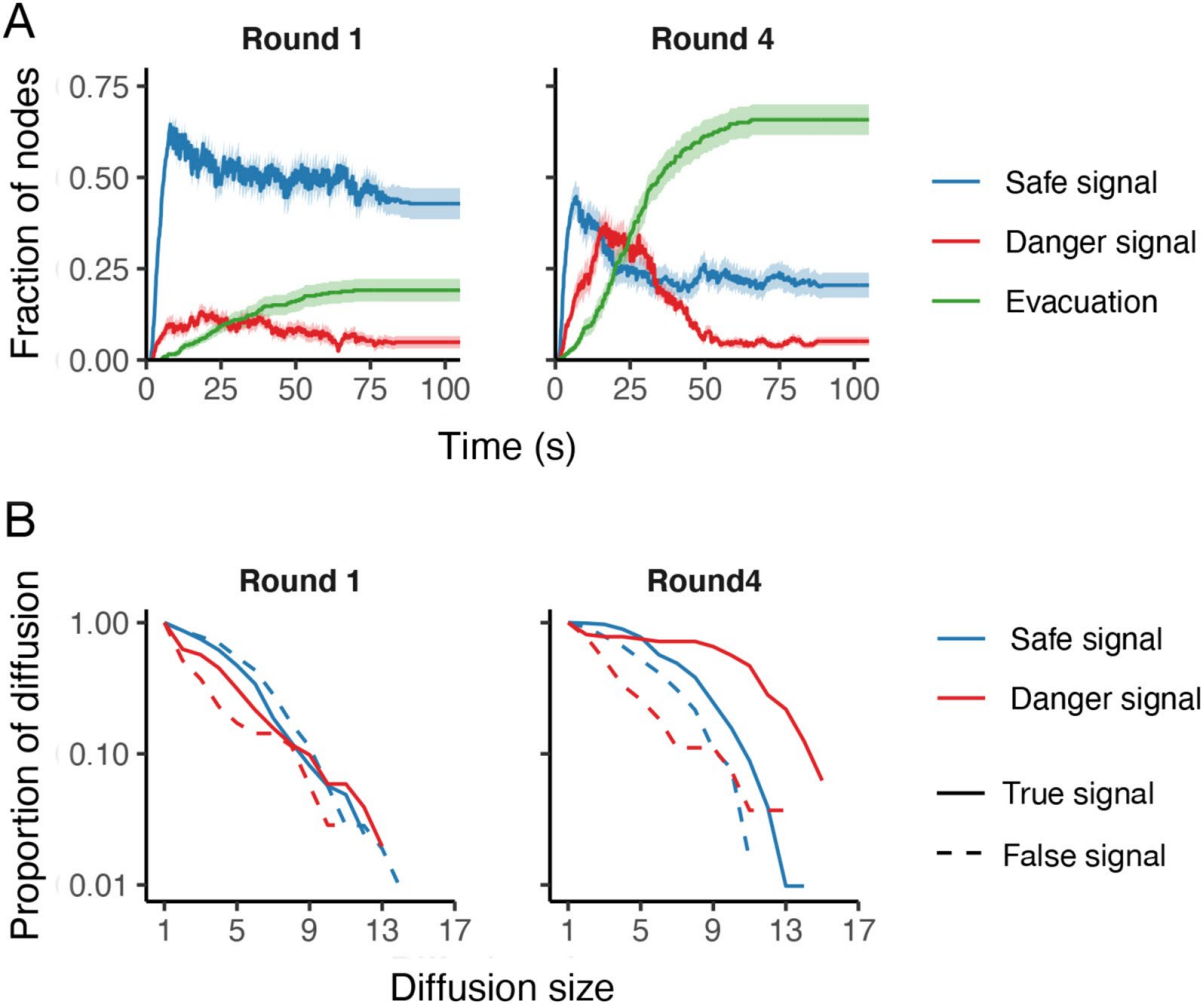
Signal diffusion changes across rounds in dynamic networks. (**A**) Average fraction of nodes showing safe signals (blue lines), danger signals (red lines), and evacuating (green lines) at the first and final rounds in the dynamic network sessions with “disaster” (*n* = 20; see Supplementary Fig. 2 for other conditions and rounds). Shading indicates 95% confidence intervals. (**B**) The complementary cumulative distribution functions of diffusions at the first and final rounds in the dynamic network sessions with and without “disaster” (*n* = 40) across signal type and accuracy (see Supplementary Fig. 3 for other conditions and rounds). Lines present the proportion of signal diffusions involving equal to or more than the number of players indicated at the x-axis. As for signal accuracy, while there is no detectable difference at round 1 (*P* = 0.839; Kolmogorov–Smirnov test), the diffusion size significantly varies at round 4 (*P* < 0.001; Kolmogorov–Smirnov test).

The improvement in necessary evacuations within networks is associated with corresponding changes to the signal diffusion among individuals (Fig. 2A). At round 1, false safe signals constantly overwhelmed true warnings for the entire time, even when an informant was informed of the impending danger. However, the diffusion dynamics changed as the rounds progressed. At round 4, false safe signals surged as with round 1, but true warnings also spread immediately after safe signals, and mass evacuation followed (Fig. 2A). The surge of warnings was more prominent in the dynamic network condition than the static network condition (Supplementary Fig. 2).

To examine the change in signal diffusion, I analyzed “diffusion chains” for each signal type in the network sessions (see “Methods”). Figure 2B shows the complementary cumulative distributions of diffusion chains by size at rounds 1 and 4. At round 1, the signal’s diffusion size does not vary by the signal’s accuracy (*P* = 0.969 in the static network condition; *P* = 0.839 in the dynamic network condition; Kolmogorov–Smirnov test). At round 4, however, the signal’s accuracy made a significant difference (*P* < 0.001 in both network conditions; Kolmogorov–Smirnov test), and the difference was more extensive than that in signal type. The difference was prominent especially in dynamic networks (Supplementary Fig. 3).

How could people improve collective performance in signal diffusion across rounds, especially when disaster would strike? Theory suggests two nonexclusive mechanisms for improving communication quality: *individual learning* and *network evolution*^12,19^. To examine the mechanisms, I conduct three analyses. First, I analyze the individual-level change in probabilities of response to neighboring signals. Then I investigate the impact of network structure on evacuation performance compared with the supplementary condition of random informants. Finally, I examine the isolated effect of network structure controlling individual experience in the game using the isomorphic networks with additional subjects.

### Individual responsiveness and network amplification

**Figure 3:**
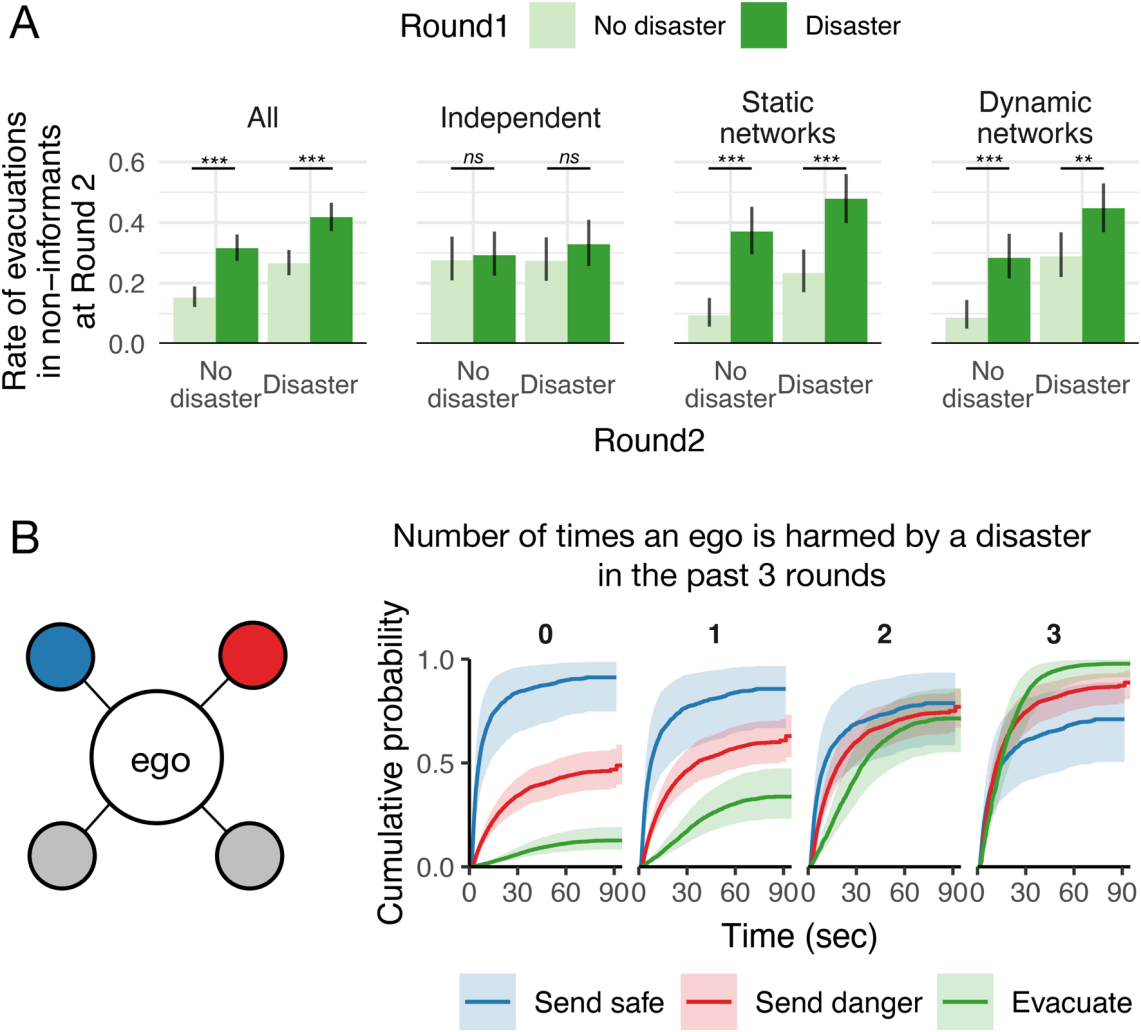
“Disaster” experience increases individual responsiveness. (**A**) The rate of evacuations in non-informants at the second round across “disaster” events at the first round. Error bars are 95% confidence intervals with Wilson method (*n* = 429.3 (s.d. = 3.5) for all the conditions, *n* = 145.3 (s.d. = 1.7) for the independent condition, *n* = 142.8 (s.d. = 4.9) for the static-network condition, *n* = 141.3 (s.d. = 1.7) for the dynamic-network condition). *ns* indicates *P* ≥ 0.05, ** indicates *P* < 0.01, *** indicates *P* < 0.001 with two proportion *z*-test. (**B**) Lines present the estimated probability of taking action at a given time if a player continues to receive the signal combination from neighbors shown in the left diagram (a player indicated by “ego” follows four other players; one neighbor keeps showing safe, another showing danger, and the other two showing no signals). Shading indicates 95% confidence intervals among individuals. The cumulative probabilities depend on how many times the player has been affected by a “disaster” in the past three rounds. As individuals experience “disaster,” they increase the probability of sending a warning and evacuating upon exposure to more warnings than safe messages. The neighborhood sample is arbitrarily selected to show the changes in individual responsiveness while controlling for signal exposure from neighbors. See the statistical models and results in “Methods” and Supplementary Table 4.

For the first mechanism analysis, I used the variation of “disaster” events in the initial two rounds to confirm that subjects’ prior experiences affected evacuation decision-making (Fig. 3A). When they experienced a “disaster” at the first round, they were more likely to evacuate in both “no disaster” and “disaster” sessions at the second round (*P* < 0.01 for both “no disaster” and “disaster” sessions of all at round 2; two proportion *z*-test). The effect of prior “disaster” experiences was statistically significant in the network sessions (*P* < 0.01 for all the combinations of “no disaster” and “disaster” sessions and static network and dynamic network conditions), but not in the independent sessions (*P* = 0.85 for “no disaster” sessions; *P* = 0.38 for the “disaster” sessions of the independent condition).

To examine the details of the contextual effect, I estimated the cumulative probability of the subject’s actions over time depending on individuals’ prior experience of “disasters” (Fig. 3B). For the estimation, I used Cox regression models to control time-varying signal influence from neighbors (Supplementary Table 4; see “Methods” for details). As with prior work^23^, subjects express safe signals more than danger signals as a default (regardless of actual “disaster” situations). However, as individuals experience a “disaster” and its damage, their responsiveness changes significantly; they are more likely to send a danger signal and evacuate than send a safe signal even upon the same signal exposure. When most subjects have changed their responsiveness through repeated failures in evacuation, a warning is likely to spread further than a safe signal when needed (Fig. 2).

### Network structure and effective tie rewiring

The individual-level analysis also shows that the effect of network dynamics shown in Fig. 1 is entirely mediated by individual exposure (Supplementary Table 4). Hence, network dynamics alter an individual’s signal exposure and ultimately evacuation behavior without subjects changing their behavior as individuals. In other words, the dynamic network setting helped people evolve their network to be more effective in spreading true signals.

Then, how could tie rewiring help people make their network more effective in spreading true signals? In the dynamic network condition, subjects updated their connections based on the last-round information regarding the other player (“counterpart”), such as whether the counterpart had evacuated and how long they had sent each signal. The information significantly affected their tie-rewiring decisions (Supplementary Fig. 4A). The average percentage at which a subject made a tie with a counterpart who had taken correct action is 77.8%, and the average percentage at which a subject broke a tie with them is 23.0%. Otherwise, subjects made a tie at a rate of 50.6% and broke a tie at a rate of 48.9%. The tie-rewiring decisions by signal information show a similar trend (Supplementary Fig. 4B).

Subjects in the additional sessions with random informants display almost the same tie-rewiring heuristics (Supplementary Fig. 4). As a result, the simple structural features of a network—such as density, average path length, and clustering coefficient—had no significant difference between these fixed-informant and random-informant sessions (Supplementary Fig. 5B). However, this random informant setting decreased the improvement in collective performance (Supplementary Fig. 5A). Hence, the increase in network connections and the reduction in path length did not cause the improvement of information sharing and collective performance with network dynamics in this experiment.

**Figure 4:**
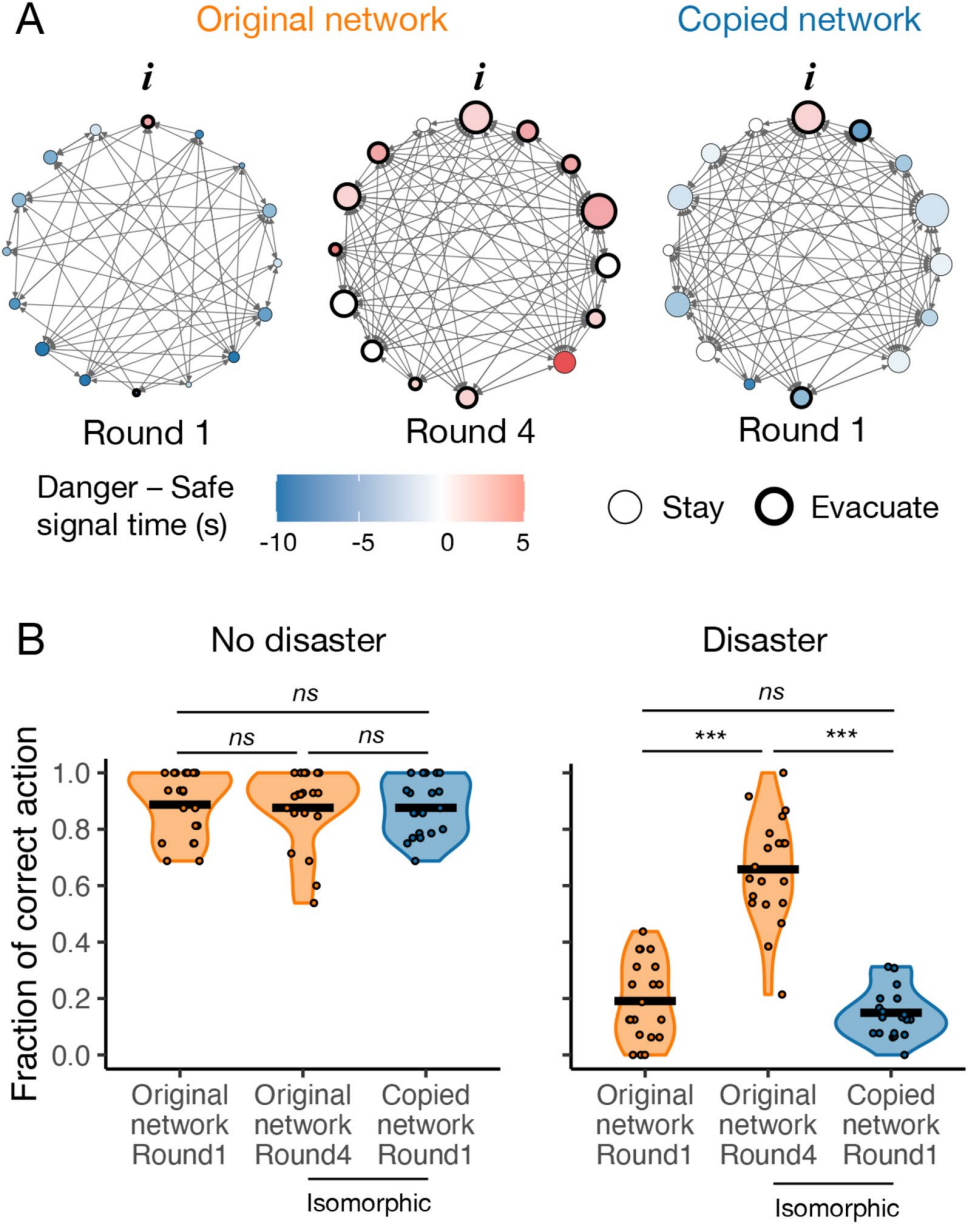
Isomorphic networks do not improve collective performance without individual experience. (**A**) The states at the first and final rounds in an example session of the dynamic network condition with always “disaster” occurrence, and the state at the first round with the isomorphic network of the original session at the final round. Node position fixes with individuals in the original session. Node size indicates in-degree (the number of connections pointing to the node; i.e., the number of people “listening” to the player). Node color indicates the signaling time of safe and danger signals. The bold outline frame indicates the subject’s evacuation. “i” indicates the node of an informed subject. (**B**) Behavioral accuracy in dynamic networks at the first and final rounds, compared with copied, isomorphic networks at the first round. Dots show results for each session, shadings show the distributions of them, and black crossbars show the averages (*n* = 20). *ns* indicates *P* ≥ 0.05 and *** indicates *P* < 0.001 with *t* test. Without individual experience, network structure alone does not improve behavioral accuracy in the sessions with “disaster”.

A clear structural difference made by the informant setting is in-degree (i.e., the number of connections pointing to the node) between informants and non-informants (Supplementary Fig. 5C). In the random-informant condition, the in-degree of informants was on average the same as that of non-informants. Informants had no distinctive influence from the structural perspective. By contrast, in the fixed-informant condition (i.e., the main dynamic network condition), informants attracted significantly more connections than non-informants across rounds. As a result, more subjects could receive signals directly from informants (who had the true information about “disaster”) (Fig. 4A). The structural difference was also directed; out-degree (i.e., the number of connections pointing out of the node) had no difference between informants and non-informants (Supplementary Fig. 5C). Others’ past performance was meaningful feedback to assess their future performance only when information sources remained the same.

### Isolated effects of network structure

Finally, I examined the isolated effect of network structure that subjects developed in the dynamic network condition. In a supplementary experiment involving 567 additional, inexperienced subjects and a matched set of 40 graphs, they played a single-round evacuation game in the same network topology, informant’s node, game length, and disaster occurrence as other subjects had played at round 4 in the dynamic network condition (Fig. 4A).

I found that network structure *alone* did not contribute to the improvement in collective intelligence shown in the dynamic networks. Figure 4B shows that, in the sessions with disasters, the first-round performance of subjects interacting in the copied, isomorphic networks was significantly lower than the fourth-round performance of subjects interacting in the networks that they developed on their own (*P* < 0.001; paired *t* test). Furthermore, there was no difference from the sessions of original networks at the first round (*P* = 0.273; Welch two-sample *t* test), although the copied networks were denser, shorter, and more clustered, and informants were center in the networks (Fig. 4A). This finding suggests that social networks can only exert their positive structural effect on collective performance when the structure is based on the history of the individuals within them.

## Discussion

I find that dynamic social networks help people in groups improve behavioral choice accuracy in responding to an experimentally induced and uncertain danger (Fig. 1). In keeping with prior work^23^ and real emergencies^24,25^, interpersonal communication reduced needless evacuations when there was no danger, but, at the same time, it also reduced necessary evacuations when there was a danger, absent prior experience. At the first round, people in a network often displayed both procrastination and false reassurance. As they experienced “disaster” events, however, they collectively changed the pattern of signal diffusion to pass on a true warning further within the network, and they successfully increased necessary evacuation (Fig. 2). Thus, people can overcome the coordination difficulty caused by harmful social reinforcement as they experience negative outcomes and instantiate the experience in their particular connections.

The findings here suggest a possible beneficial effect of collective learning in responding to risk and uncertainty. The wisdom of crowds is optimized when decision-making is independent^43,44^. However, when people can learn from their experience with appropriate feedback, social networks can indeed improve collective intelligence with interdependence^17,18,39^. The results align with the collective encoding of risk in community ecology as well^19,20,45^. People change their perceptions about external risk factors through their own experience and reflect the updated views in interpersonal communication (Fig. 3). As a result, they can increase risk awareness as a group^46^. This also suggests that network-based social intelligence may depend on environmental and historical contexts^35^. A current group’s success could arise from past challenging experiences and collective efforts.

As with prior work^23^, most players did not evacuate at the first round in all the conditions (Fig. 1C). In addition, players expressed safe signals more than danger signals in the network sessions even with a “disaster” (Fig. 2A). The game’s reasonably asymmetric nature between the behavioral options can cause the bias toward the status quo and consequently the risky choice (i.e., not to evacuate). In contrast to the standard risky-choice task (where people choose from risky and safe options presented simultaneously^39,40^), subjects here were asked whether to take risk-avoiding-but-costly action, and they could decide it anytime within an allotted time. Subjects with an endowment at the outset of a game could be unwilling to trade it for their security in conditions of ignorance or uncertainty^47^. As an alternative explanation, subjects might underestimate the “disaster” risk even though they were informed that “a disaster may or may not strike” because extraordinary accidents rarely happen in real life. Also, subjects might find it easy to try the risky choice with the small economic stakes at the first round of the iterated game. In the network condition, some (uninformed) subjects could send safe signals easily with their initial belief of no “disaster,” lacking a long-term horizon. On the other hand, some subjects could hesitate to issue warnings because they were afraid of inducing unnecessary confusion and loss (by evacuation when a disaster eventually did not materialize)^48^. Because warnings are valuable but *undesirable* messages, as a default, uninformed individuals amplify popular taste and refuse to listen to unpopular opinions from the minority (i.e., the informant)^23,49^. Thus, this study examines how a group of people becomes able to defy internal inertia in repetitive responses to external threats.

In this experiment, the simple structural features of a network—such as density, average path length, and clustering coefficient—made little difference in collective performance (Supplementary Fig. 5). This null result suggests that information and behavior do not spread with single events, like germs, especially when accuracy matters^37^. In such a situation, people often aggregate information to assess what is happening^25^. However, interpersonal signals vary significantly in their veracity^4^. Thus, people having more connections to others are not only more advantaged for access to the truth, but they are also more vulnerable to falsehoods. This experiment suggests that pure topological features may not contribute to collective learning, especially in adverse conditions.

Instead, social networks need to put the right people (i.e., those who take appropriate behavior in context) in the right place (i.e., high in-degree nodes) to facilitate collective intelligence in circumstances involving the admixture of truth and falsehood^4,11^. People tried to align their network position to their individual behavior in dynamic networks (Supplementary Fig. 4), and the structural adaptation made a significant difference in collective performance (Fig. 1), but only when the same people took on the role as information sources across rounds (Supplementary Fig. 5).

This study also shows that the emerging topological pattern of the developed networks is effective only when combined with individual responsiveness (Fig. 4). Network structure and dynamics might have little effect on collective performance when individual experience and accumulated relationships are not incorporated into them^30^. These findings suggest the importance of clarifying conditions for social networks to facilitate collective learning through network connections and adaptation^50^. Information sources and their centrality command attention, but the rest of the population, i.e., uninformed individuals and their responsiveness also matter^49,51^.

It is important to note that simple, random play is not compatible with the experimental results. The spreading of safe and danger signals would have occurred at the same frequency if subjects randomly sent signals without understanding the payoff difference. Furthermore, the overall accuracy would have not improved (i.e., no learning effect) if subjects evacuated simply by lapse of time because the percentage of correct actions would decrease in the no-disaster rounds as well as that would increase in the disaster rounds. Mere spontaneous drift also cannot explain the better improvement in the dynamic-network sessions, compared to the static-network and independent ones.

Although the results of laboratory experiments do not translate directly into the real world, the evidence presented here suggests that dynamic social networks might help people to turn group failure into success in uncertain or dangerous situations. With the growing dependence on personal communication channels and their widening scale, the negative aspects of network diffusion have received considerable attention^21^. Deepening the understanding of social networks as means of positive collective learning might help us to defuse worrisome social contagions.

## Methods

### Experimental setup

This research was approved by the Carnegie Mellon University Committee of the Use of Human Subjects. All methods were performed in accordance with the relevant guidelines and regulations. Informed consent was obtained from all participants. Our data includes no identifying information of human participants. We conducted experiments from February to August 2021 (except for the preliminary sessions of random information; we ran the condition from June to November 2020). We preregistered the main experiment settings using AsPredicted (https://aspredicted.org/sm4k5.pdf).

A total of 2786 subjects participated in our incentivized decision-making game experiments. We recruited subjects using Amazon Mechanical Turk (MTurk)^52,53^. Supplementary Table 1 shows the subject demographics. Our participants interacted anonymously over the Internet using customized software playable in a browser window (available at http://breadboard.yale.edu). All participants provided explicit consent and passed a series of human verification checks and a screening test of understanding game rules and payoffs before playing the game (see SI). We prohibited subjects from participating in more than one session of the experiment by using unique identifications for each subject on MTurk.

In each session, subjects were paid a $2.00 show-up fee and a bonus depending on whether they took the appropriate disaster decision in four rounds. Furthermore, subjects earned $1.00 when they completed all four rounds. In each round, when a disaster stroke before they evacuated, the subjects earned no bonus. Otherwise, they earned a bonus of $1.00 without disaster or $0.50 with disaster by spending $0.50 for evacuation, plus $0.05 per other players who took the correct action accordingly (Supplementary Table 2). We have confirmed with prior work that the amount of evacuation cost, if any, makes no significant difference in the game’s performance^23^.

At the start, subjects were required to pass a series of human verification checks. They needed to pass Google’s reCAPTCHA using the “I’m not a robot” checkbox. They were also requested to answer whether they were human players. The exact question asked was: “Please select an applicable answer about you.” The options were: “I am not a bot. I am a real person.” “I am not a real person. I am a bot.” “I am anything but a human.” and “I am a computer program working for a person.” The option’s order was randomized. Only the participants who selected “I am not a bot. I am a real person.” moved to the step of informed consent.

When subjects provided explicit consent, they were asked to take a tutorial before the actual game would begin. In the tutorial, each subject separately interacted with three dummy players in two rounds of a 45-s practice game. In the actual game, some subjects would be informed in advance whether a disaster would indeed strike or not. In the practice game, while all subjects were not informed of such information in the first round, they were informed of the information in the second round. Thus, they practiced both conditions in terms of prior information on the disaster (see SI).

After the practice game, subjects were assessed for their comprehension of the game rules and payment structure using four multiple-choice questions with three options. If they failed to select the correct answer in one of the questions, they could reselect it only once through the entire test. If they failed to select the correct answer more than once, they were unable to join the actual game.

At 720 s after the tutorial beginning, a “Ready” button became visible simultaneously to all the subjects who completed the tutorial and passed the comprehension tests. The actual games started 30 s after the “Ready” button showed up. If subjects did not click the button before the game started, they were dropped. The game required a certain number of subjects. When the subjects who successfully clicked the button were more than 16, surplus subjects, randomly selected, were dropped from the game. When the number of qualified subjects was less than 12, the game did not start. As a result, subjects started the game in a group with an average size of 15.5 (s.d. = 1.1).

At the start of the actual game, we selected one subject (the “informant”) at random who was informed in advance whether a disaster would indeed strike or not. The other subjects were informed that some players had accurate information about the disaster, but they were not informed who the informant was. The exact sentence that the informants received in their game screen was “A disaster is going to strike!” when a disaster would strike or “There is no disaster.” when a disaster would not strike. The one that the other uninformed subjects received was “A disaster may or may not strike.” Then, the group had the same informant across the four rounds except for a supplement condition of random informants. In the random informant condition, an informant was randomly selected every round.

To prevent an end-of-game effect, we randomly set the game time with a normal distribution of a mean of 75 s and a standard deviation of 10 s. Prior work has confirmed that the game time is sufficient for players to communicate and make an evacuation decision^23^. As a result, each round ended at 75.0 s on average (s.d. = 9.5) without prior notice. In half of the sessions, a disaster struck at the end of the game. We did not inform any subjects, including the informants, when their sessions would end, the global network structure they were embedded in, or how many informants were in the game. After making their evacuation choice, subjects were informed of their success and failure along with overall results in their group. Then, subjects played another round of the evacuation game until they completed four total rounds. They had the same local network environment across four rounds except for the dynamic network condition.

### Network structure and tie rewiring

In the network sessions, subjects played the game in a directed network with a random graph configuration. A certain number of ties were present at the game’s onset as the initial density was set to 0.25.

In the dynamic network conditions, subjects also could change their neighbors by making or breaking ties between rounds. In the tie-rewiring step, 40% of all the possible subject pairs were chosen at random. Thus, subjects could choose every other player at least once throughout the entire session (i.e., a set of four rounds) with a probability of about 80%. When the chosen pairs were connected, the pairs (the ties) were dissolved if the predecessor subject of the directed ties chose to break the tie. When the chosen pairs were not connected, the pairs (the ties) were newly created when the predecessor of the potential tie chose to create the tie. Subjects were not informed of the rewiring rate.

To equalize the game time, we made subjects in the independent and static network conditions wait for additional 10 s after each game round ended. Despite the adjustment, the game time was significantly longer in the dynamic network sessions than in the independent and static network sessions. The average game time is 429.5 s (s.d. = 20.2) for the independent condition; 428.8 s (s.d. = 19.0) for the static network condition; and 564.7 s (s.d. = 36.3) for the dynamic network condition.

To clarify mechanisms for dynamic networks to facilitate collective intelligence, we added one supplementary condition. In the supplementary condition, subjects were assigned to one of the 40 isomorphic networks that other subjects had developed with tie-rewiring options through the three rounds in the dynamic network condition (567 subjects in 40 groups). Network structure and other game settings (i.e., whether a disaster stroke, how long the game was, and which node was the informant) were identical to where the others played the game at the final round. However, players were different, and they had no prior experience in the game. They played the game in a network with a topology created by others ostensibly to optimize the accurate flow of information. In contrast to other conditions, subjects played only one round in the isomorphic network condition.

### Signal buttons

During the game of network sessions, subjects were allowed to share information about the possibly impending “disaster” by using “Safe” and “Danger” buttons that indicated their assessment (see SI). The default node color was grey. Then, when they clicked the Safe button, their node turned blue and, after 5 s, automatically returned to grey. Likewise, the Danger button turned their node to red for 5 s. Subjects could see only the colors of neighbors to whom they were directly connected. Since the signal exchange occurred through directed connections, an individual could send, but not receive, information from another subject (and vice versa). Once subjects chose to evacuate, they could no longer send signals, and their node showed grey (the default color) for the rest of the game. The neighbors of evacuated subjects were not informed of their evacuation. We have confirmed with prior work that collective performance does not vary with the communication continuity and the evacuation visibility^23^. Subjects could use the Safe and Danger buttons any time unless they evacuated, or they did not have to.

### Players dropping during the game

After each game round, when a player was inactive for 10 s, they were warned about being dropped. When they remained inactive after 10 s, they were dropped. When the selected informant was dropped, the session stopped at the round, and we did not use the data. Furthermore, as too many dropped players could affect the network structure and the behavioral dynamics of remaining players, we did not use the sessions where more than 25% of initial players were dropped during the game. Overall, 4 players dropped in 15 sessions; 3 players dropped in 22 sessions; 2 players dropped in 41 sessions; 1 player dropped in 44 sessions; and no player dropped in 58 sessions. The dropped players were prohibited from joining another session of this experiment.

As noted above, players took the additional tie-rewiring step every round in the dynamic network sessions. Thus, the total game time was longer in the dynamic network sessions than in the independent and static network sessions even with the adjustment. As a result, more players were dropped in the dynamic network sessions than in the independent and static network sessions. The average number of dropped players across the four rounds is 0.40 (s.d. = 0.60) for the independent condition; 1.15 (s.d. = 0.86) for the static network condition; 1.75 (s.d. = 1.19) for the dynamic network condition. Although group size could affect collective performance, we found the differences in group size small enough for our study. We have confirmed the dynamic network's performance improvement with a comprehensive analysis controlling the effect of group size (Supplementary Table 3). Also, there was no statistically significant difference in the dropped players’ performance of the dynamic network condition, compared with the other two conditions. The rate of correct actions of dropped players is 0.456 (s.d. = 0.322) for the independent condition, 0.594 (s.d. = 0.387) for the static network condition, and 0.558 (s.d. = 0.411) for the dynamic network condition; *P* = 0.106 between the independent condition and the dynamic network condition; *P* = 0.599 between the static network condition and the dynamic network condition (Welch two-sample *t* test).

### Analysis of signal diffusions

To examine the change in signal diffusion, we analyzed “diffusion chains” for each signal type in the network sessions. We first identified the subjects who sent a signal when their neighbors had never sent one as spontaneous “diffusion sources.” When a subject sent a signal after at least one neighbor had sent the same type of signal, we regarded the subject’s signaling (and evacuation with danger signals) as occurring in a chain of signal diffusion and the total number of the responded subjects (including the diffusion source) as the diffusion size.

We analyzed the distribution of signal diffusion chains with complementary cumulative distribution functions, measuring the fraction of diffusion chains that exhibit a given number of diffusion sizes. We found that the number of diffusions of both signals did not change across rounds. Safe-signal diffusions were more likely to occur than danger-signal diffusions regardless of whether a “disaster” would strike and how many rounds subjects played. On the other hand, the diffusion size varied greatly across rounds in disaster situations. With “disaster,” false safe signals spread further than true danger signals at the first round, but after that, warnings outperformed safe signals in terms of diffusion size. Figure 2B and Supplementary Fig. 3 scrutinize the changes in diffusion chains with their distributions.

### Analysis of individual responsiveness

We analyzed how individual evacuation behavior varies with exposure to signals from neighbors^54^. Let

$${a}_{i}^{evacuate}\,\, (t)=\left\{\begin{array}{ll}1&\quad \text{if subject } i \text{ evacuates at time } t\\ 0&\quad \text{otherwise}\end{array}\right.$$

$${a}_{i}^{show\, safe}\,\, (t)=\left\{\begin{array}{ll}1&\quad \text{if subject } i \mathrm{ shows a safe signal at time } t\\ 0&\quad \text{otherwise}\end{array}\right.$$

$${a}_{i}^{show \, danger} \,\, (t)=\left\{\begin{array}{ll}1&\quad \text{if subject } i \text{ shows a danger signal at time } t\\ 0&\quad \text{otherwise}\end{array}\right.$$

The hazard function, or instantaneous rate of occurrence of subject $i$’s evacuation at time *t,* is defined as:

$${\lambda }_{i}\,\, (t)=\underset{\mathit{dt}\to 0}{{\mathrm{lim}}}\frac{{\mathrm{Pr}}({a}_{i}^{evacuate}=1;\,\, t<T\le t+dt|T>t)}{dt}$$

To model the time to evacuation, We used a Cox proportional hazards model with time-varying covariates for the number of signals, incorporating an individual actor-specific random effect^55^:

$${\lambda }_{i} \,\, \left\{t|{{P}_{i}, X}_{i}(t), {G}_{i},{Y}_{i}(t)\right\}={\lambda }_{0}(t)\mathrm{exp}\left\{{{\beta }_{P}^{{\prime}}{P}_{i}+\beta }_{X}^{{\prime}}{X}_{i}(t)+{\beta }_{G}^{{\prime}}{G}_{i}+{\beta }_{Y}^{{\prime}}{Y}_{i}(t)+{\gamma }_{i}\right\}$$

where *λ*_0_(*t*) is a baseline hazard at time *t*.

In the model, the hazard *λ*_*i*_(*t*) depends on the covariates *P*_*i*_, *X*_*i*_(*t*), *G*_*i*_, and *Y*_*i*_(*t*). The covariate *P*_*i*_ is the vector of subject *i*’s experiences before the sessions; that is, the number of rounds, the number of disasters that she has experienced, and the number of disasters that she has been struck by.

The covariate *X*_*i*_(*t*) is the vector of the number of safe signals ${x}_{i}^{safe} (t)$, the number of danger signals ${x}_{i}^{danger} (t)$. When subject *j* is a neighbor of subject *i* (i.e., $j\in {N}_{i}$), subject *i* is exposed to the signal of subject *j*, so that:

$${x}_{i}^{safe}\,\, (t)=\sum_{j\in {N}_{i}}{a}_{j}^{show\, safe}(t)$$

$${x}_{i}^{danger}\,\, (t)=\sum_{j\in {N}_{i}}{a}_{j}^{show\, danger}(t)$$

The covariate *G*_*i*_ is the vector of the properties of the network in which subject *i* is embedded, out-degree, in-degree, and a network plasticity indicator. The covariate *Y*_*i*_(*t*) is the vector of the number of the subject *i*’s actions of sending safe and danger signals before time *t*. The coefficients *β* are the fixed effects and *γ*_*i*_ is the random effect for individual *i*. We assumed that waiting times to evacuation in different actors are conditionally independent given the sequence of signals they receive from network neighbors. This model shows how the hazard of an individual’s evacuation depends on the signaling actions of others, their network position, and experience (Supplementary Table 4). We applied the same model to the first signaling behavior.

## Supplementary Information

Supplementary Information.

## Data Availability

The data and code in this manuscript are available at Mendeley Data: 10.17632/xypt92vw4h.1.
