# Supplementary material for: Individual and collective learning in groups facing danger
Source: Sci Rep. 2022 Apr 13;12:6210. doi: 10.1038/s41598-022-10255-3 (PMC9007963; doi:10.1038/s41598-022-10255-3)
Supplement: Supplementary file 1 — Supplementary Information. [file 41598_2022_10255_MOESM1_ESM.pdf]

Supplementary Information for

Individual and Collective Learning in Groups Facing Danger

Hirokazu Shirado

Correspondence to: shirado@cmu.edu

This document includes:

Supplementary Figs. 1 to 5
Supplementary Tables S1 to S4
Experiment's instruction and tutorials

Supplementary figures and tables

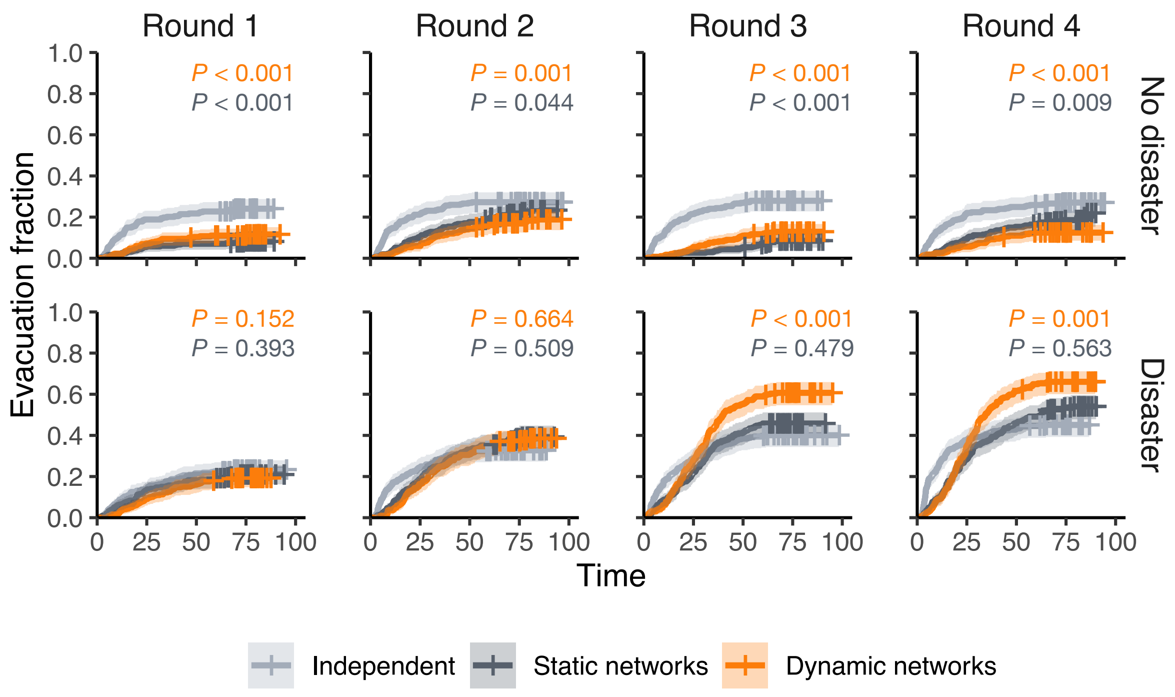

Supplementary Fig. 1.

Aggregated evacuation fraction over time. Lines indicate average fractions of evacuated subjects over time in different experimental conditions. Shades are 95% confidence intervals ($n = 20$). Censorings indicate the game's end. P-values are given by the log-rank test comparing with the results of independent sessions.

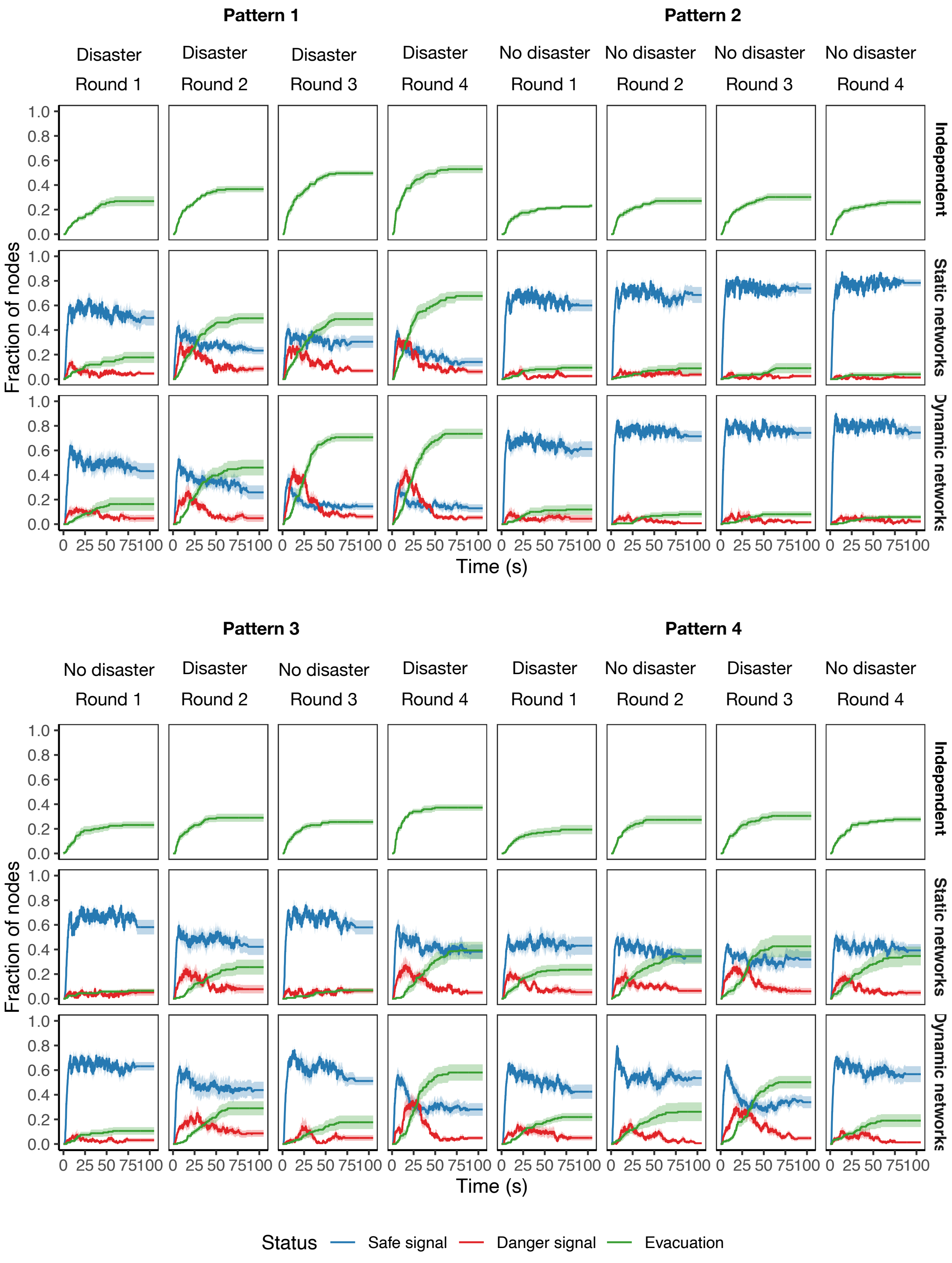

Supplementary Fig. 2.

Fraction of signals and evacuations over time across disaster patterns and network settings. Average fraction of nodes showing safe (blue lines), danger signals (red lines), and evacuations (green lines) over time. Shades are 95% confidential intervals among network sessions (n=10).

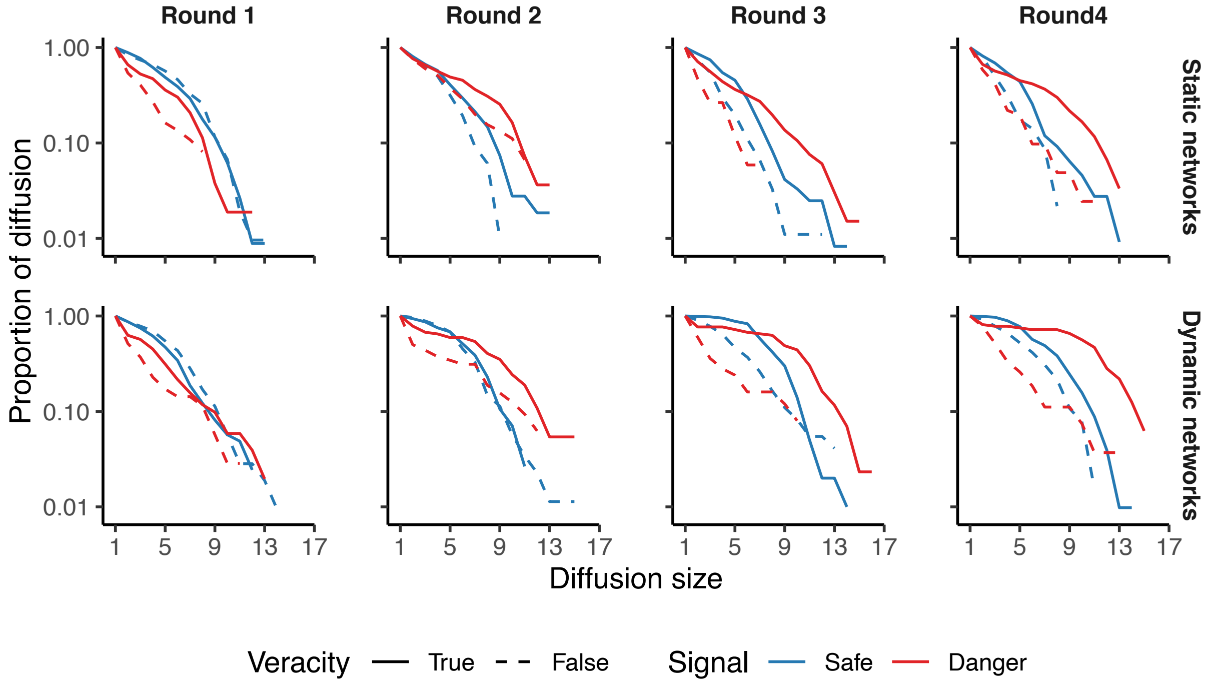

Supplementary Fig. 3.

Complementary cumulative distributions of diffusions by size across rounds in the static and dynamic network sessions. Lines present the proportion of signal diffusions involving equal to or more than the number of players indicated at the x-axis.

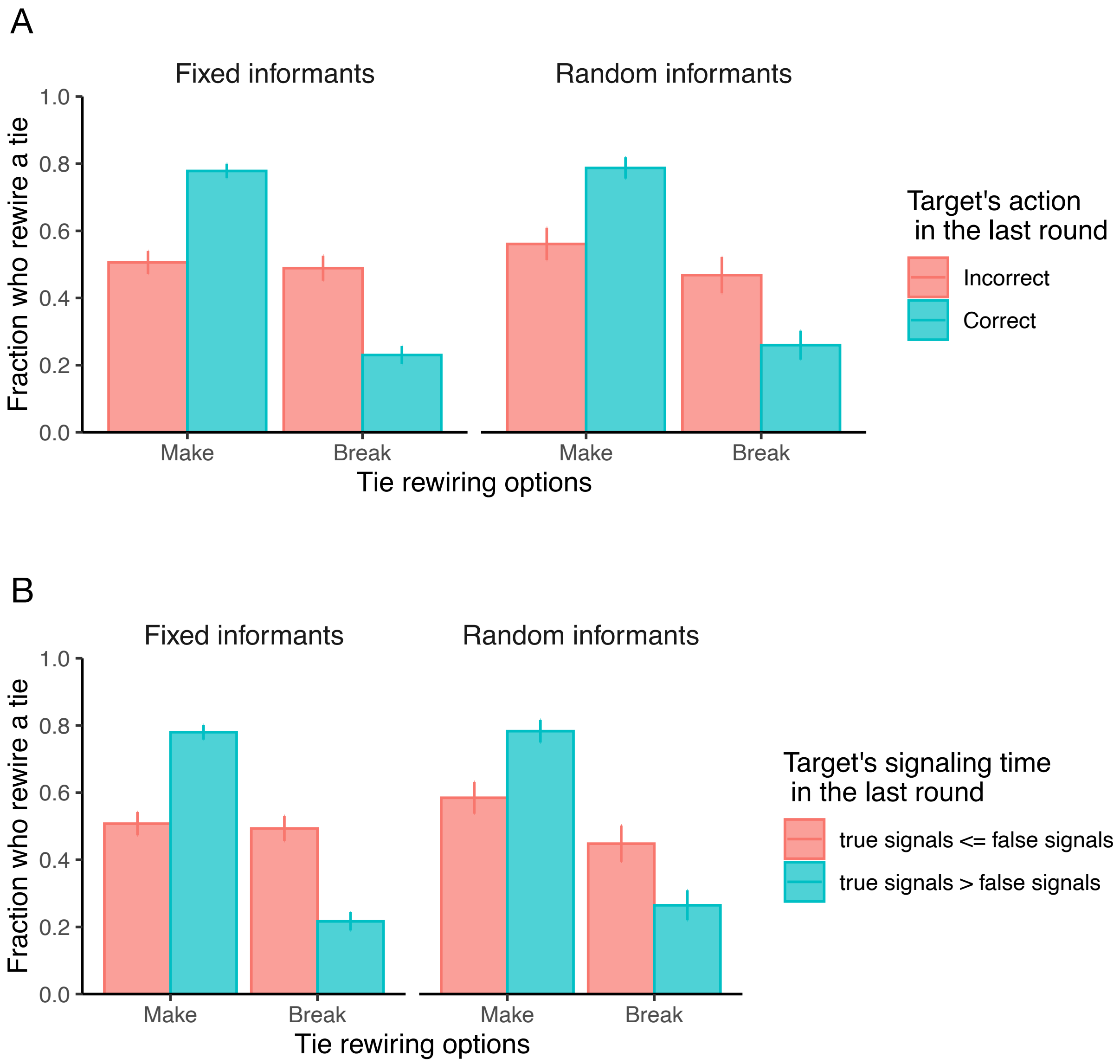

Supplementary Fig. 4.

Fraction of participants making and breaking connections across informant consistency. These results are grouped by behavior: whether the successor subject took correct action (A) and sent correct signals longer than wrong signals (B) in the previous round. All error bars are 95% confidence intervals.

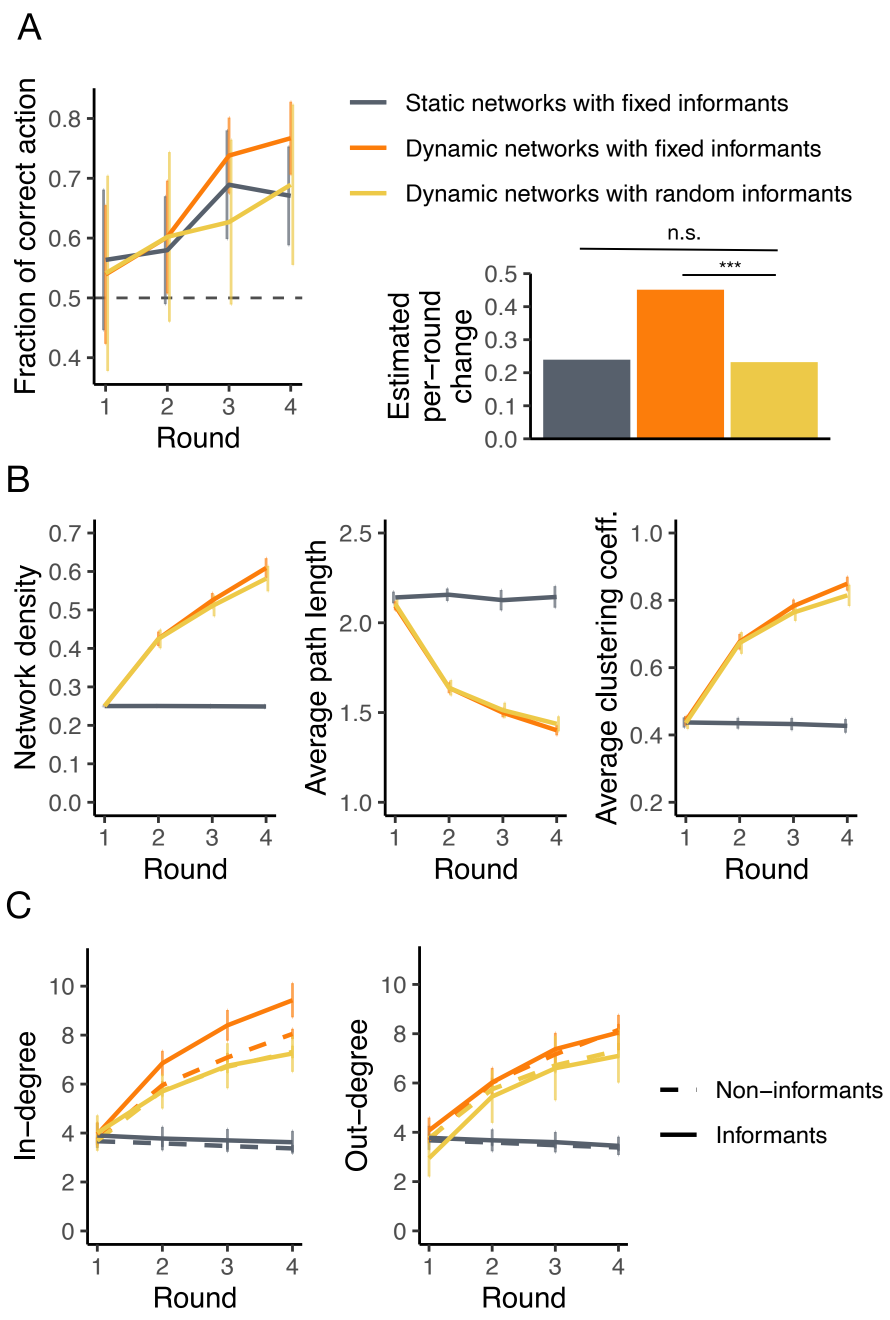

Supplementary Fig. 5.

Collective performance and network structure across informant's consistency. All error bars are 95% confidence intervals among sessions (n=40 for each condition of fixed informants; n=20 for the condition of random informants). (A) Overall behavioral accuracy over round including the sessions with random informants (indicated with yellow). The inset shows the average change in the fraction of correct action by round, estimated by GLMM using a logistic regression model. *** indicates $P < 0.01$ and n.s. indicates $P \geq 0.05$. (B) Network density, average shortest path lengths, and average clustering coefficients over round. (C) In-degree and out-degree of informants and non-informants over round.

Supplementary Table 1.

Demographics of experiment participants. The data were self-reported by the participants who completed all four game rounds (*n* = 2170).

Characteristics		Count	Percentage
Gender			
	Male	1287	59.3%
	Female	841	38.8%
	Non-binary	12	0.6%
	No answer	30	1.4%
Age			
	20 - 29	606	27.9%
	30 - 39	899	41.4%
	40 - 49	357	16.5%
	50 - 59	192	8.8%
	≥ 60	90	4.1%
	No answer	26	1.2%
Nationality			
	American	1613	74.3%
	Indian	159	7.3%
	Brazilian	85	3.9%
	Others	268	12.4%
	No answer	45	2.1%
Race / Ethnicity			
	White, Caucasian, European; not Hispanic	1536	70.8%
	Asian / Pacific Islander	278	12.8%
	Black / African american	110	5.1%
	Hipanic / Latino	122	5.6%
	American indican / Native american	24	1.1%
	Multiple ethnicity	36	1.7%
	No answer	64	2.9%
Education			
	High school or less	157	7.2%
	Some college (1-3 years)	363	16.7%
	Bachelr's degree	1135	52.3%
	Graduate degree	490	22.6%
	No answer	25	1.2%
Annual income, $			
	0 - 20,000	498	22.9%
	20,000 - 34,999	383	17.6%
	35,000 - 49,000	401	18.5%
	50,000 - 74,900	468	21.6%
	75,000 - 99,999	259	11.9%
	≥ 100,000	112	5.2%
	No answer	49	2.3%

Supplementary Table 2.

Player's payoffs per round. The value of each cell is a payoff (US$) that a subject would receive in each round. In a round, subjects receive a US$1 endowment at the outset. When they evacuate to avoid a possible disaster, they need to pay US$0.5. If a disaster strikes and subjects have not evacuated, they lose their entire endowment. Otherwise, they receive US$0.05 per another player in their group who has chosen a correct behavior (either evacuate or stay), in addition to their own leftover endowment.

		Disaster occurs	
		Yes	No
Player's choice	Evacuate	**$0.5 + 0.05\, n_{\text{Evacuate}}$** (true positives)	**$0.5 + 0.05\, n_{\text{Stay}}$** (false positives)
	Stay	**0.0** (false negatives)	**$1.0 + 0.05\, n_{\text{Stay}}$** (true negatives)

Supplementary Table 3.

The results of the statistical analysis regarding per-round changes in the fraction of correct action across network treatments, estimated by GLMM with logit model incorporating random effects for sessions.

A. Models using the independent condition as the network category's reference

	All sessions (n=120)			Sessions without disaster (n=60)			Sessions with disaster (n=60)		
	Estimated coefficient	p value		Estimated coefficient	p value		Estimated coefficient	p value	
Intercept	-0.060	0.736		1.115	0.000	***	-1.324	0.000	***
Round	0.144	0.000	***	-0.043	0.484		0.352	0.000	***
Static network (ref. Independent)	0.412	0.100		0.878	0.006	**	-0.130	0.569	
Dynamic network (ref. Independent)	0.251	0.315		0.849	0.007	**	-0.246	0.280	
Round x Static network (ref. Independent)	0.128	0.028	*	0.182	0.338		0.245	0.004	**
Round x Dynamic network (ref. Independent)	0.357	0.000	***	-0.079	0.111		0.516	0.000	***
Group size†	0.104	0.046	*	0.087	0.332		0.131	0.044	*
Game length†	-0.034	0.257		0.071	0.170		0.030	0.491	

† The covariates are standardized for estimation convergence.

* P < 0.05; ** P < 0.01; *** P < 0.001

B. Models using the dynamic network condition as the network category's reference

	All sessions (n=120)			Sessions without disaster (n=60)			Sessions with disaster (n=60)		
	Estimated coefficient	p value		Estimated coefficient	p value		Estimated coefficient	p value	
Intercept	0.192	0.292		1.964	0.000	***	-1.570	0.000	***
Round	0.502	0.000	***	0.139	0.170		0.868	0.000	***
Independent (ref. Dynamic network)	-0.251	0.315		-0.849	0.007	**	0.246	0.280	
Static network (ref. Dynamic network)	0.161	0.525		0.028	0.932		0.116	0.616	
Round x Independent (ref. Dynamic network)	-0.357	0.000	***	-0.182	0.111		-0.516	0.000	***
Round x Static network (ref. Dynamic network)	-0.230	0.000	***	-0.079	0.510		-0.272	0.002	**
Group size†	0.104	0.046	*	0.087	0.332		0.131	0.044	*
Game length†	-0.034	0.257		0.071	0.170		0.030	0.491	

† The covariates are standardized for estimation convergence.

* P < 0.05; ** P < 0.01; *** P < 0.001

Supplementary Table 4.

The results of the statistical analysis regarding whether to evacuate and whether to send the first signal (safe/danger), estimated by Cox proportional model with time-varying covariates of neighbor's signals and subject's past experiences incorporating the random effect for individuals. The models control the repeat count of the subject's past signaling.

	Evacuate			Send the first safe signal			Send the first danger signal		
	Hazard ratio	P value		Hazard ratio	P value		Hazard ratio	P value	
Ego's past experience									
Num. round†	1.042	0.510		1.177	0.000	***	0.961	0.390	
Num. disaster†	1.095	0.120		0.679	0.000	***	1.139	0.005	**
Num. disaster affected†	1.709	0.000	***	1.109	0.030	*	1.142	0.002	**
Signal exposure from neighbors									
Rate of safe signals†	0.569	0.000	***	1.415	0.000	***	0.727	0.000	***
Rate of danger signals†	1.332	0.000	***	0.559	0.000	***	1.675	0.000	***
Network property									
Out degree†	1.151	0.021	*	1.120	0.001	**	1.206	0.000	***
In degree†	0.998	0.970		0.990	0.790		1.010	0.850	
Dynamic network (ref. Static network)	1.159	0.320		1.035	0.710		0.849	0.120	
Ego's other actions before the focal action									
Num. sending safe signals†	0.107	0.000	***				0.328	0.000	***
Num. sending danger signals†	1.201	0.000	***	0.233	0.000	***			

† The covariates are standardized for estimation convergence.

* $P < 0.05$; ** $P < 0.01$; *** $P < 0.001$

Experiment's instruction and tutorials

Below are screenshots for the initial description of the tutorial and the confirmation tests. We also show example screenshots of the actual game. These screenshots are about the dynamic network condition. In the independent and static network conditions, there were no rewiring steps and tutorials about them.

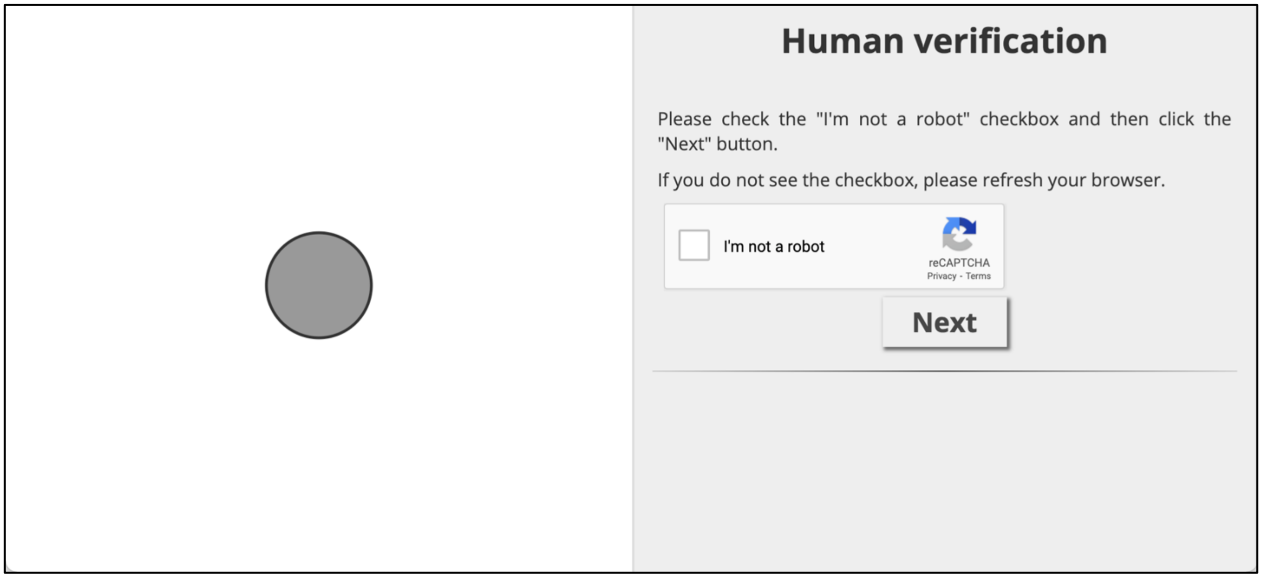

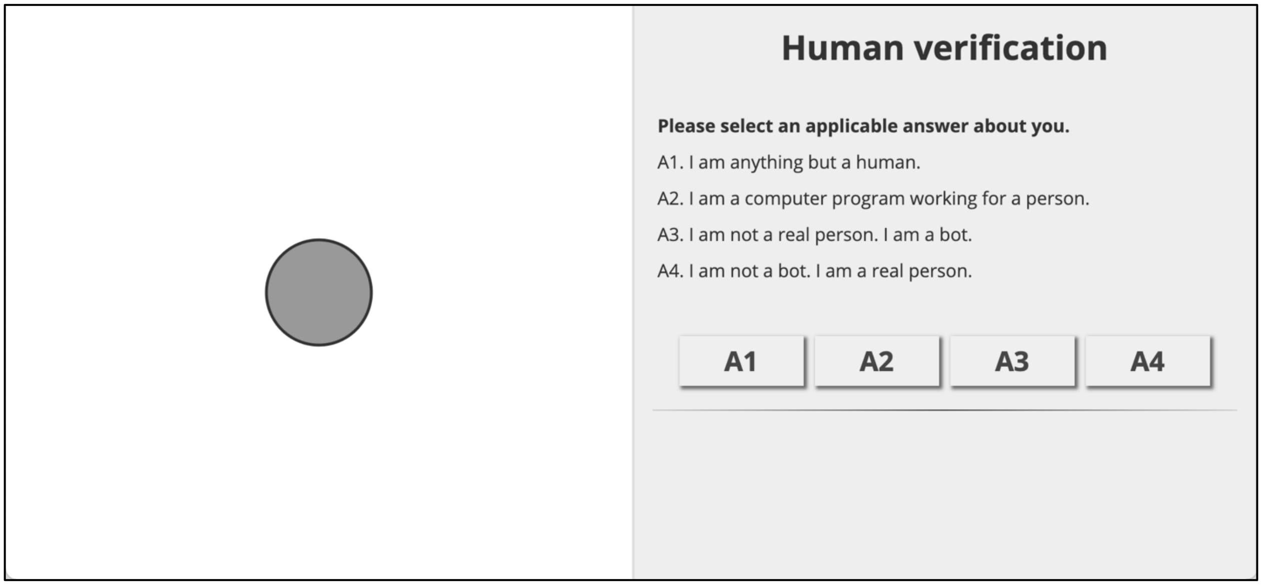

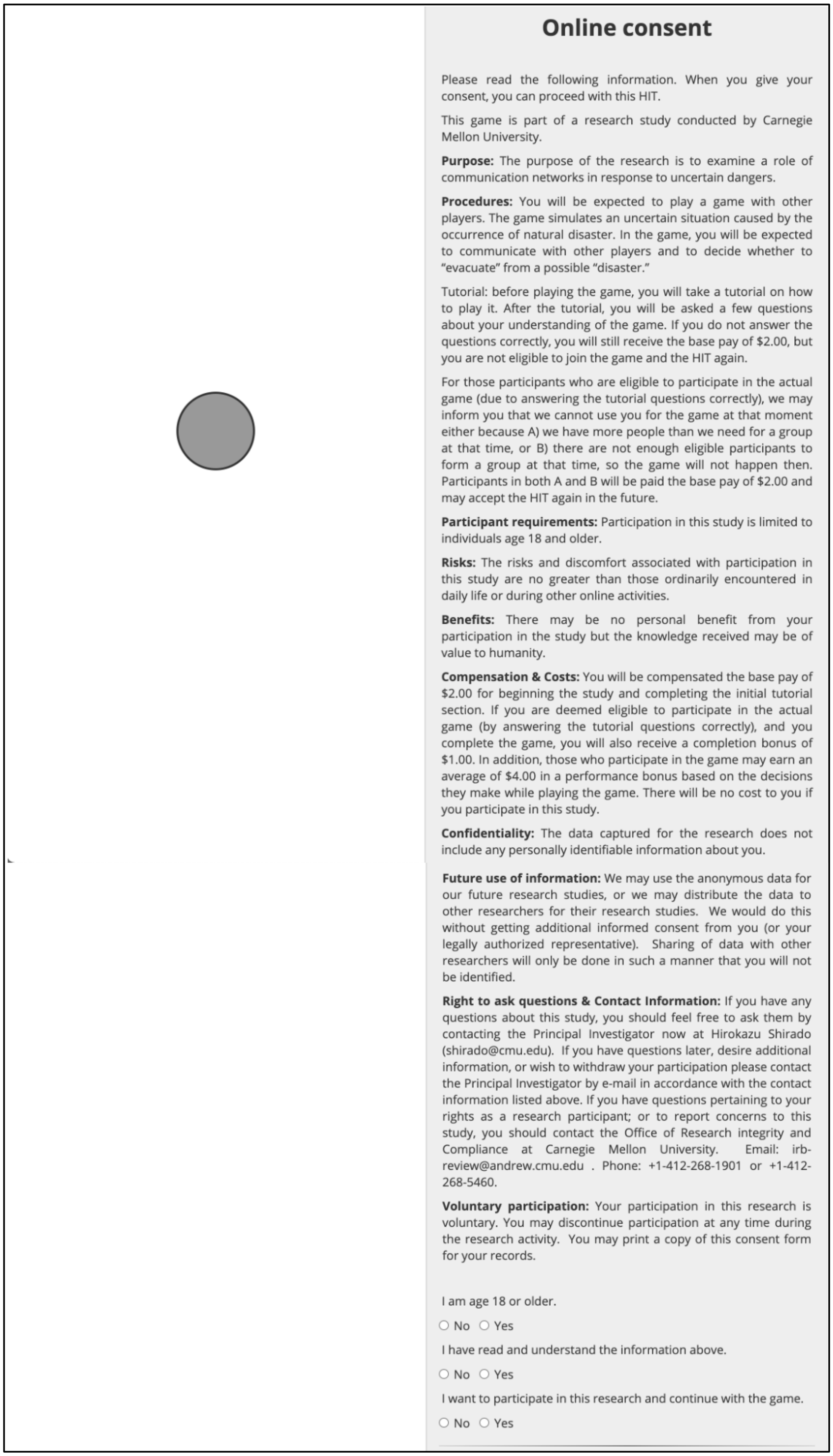

Online consent

Please read the following information. When you give your consent, you can proceed with this HIT.

This game is part of a research study conducted by Carnegie Mellon University.

Purpose: The purpose of the research is to examine a role of communication networks in response to uncertain dangers.

Procedures: You will be expected to play a game with other players. The game simulates an uncertain situation caused by the occurrence of natural disaster. In the game, you will be expected to communicate with other players and to decide whether to "evacuate" from a possible "disaster."

Tutorial: before playing the game, you will take a tutorial on how to play it. After the tutorial, you will be asked a few questions about your understanding of the game. If you do not answer the questions correctly, you will still receive the base pay of $2.00, but you are not eligible to join the game and the HIT again.

For those participants who are eligible to participate in the actual game (due to answering the tutorial questions correctly), we may inform you that we cannot use you for the game at that moment either because A) we have more people than we need for a group at that time, or B) there are not enough eligible participants to form a group at that time, so the game will not happen then. Participants in both A and B will be paid the base pay of $2.00 and may accept the HIT again in the future.

Participant requirements: Participation in this study is limited to individuals age 18 and older.

Risks: The risks and discomfort associated with participation in this study are no greater than those ordinarily encountered in daily life or during other online activities.

Benefits: There may be no personal benefit from your participation in the study but the knowledge received may be of value to humanity.

Compensation & Costs: You will be compensated the base pay of $2.00 for beginning the study and completing the initial tutorial section. If you are deemed eligible to participate in the actual game (by answering the tutorial questions correctly), and you complete the game, you will also receive a completion bonus of $1.00. In addition, those who participate in the game may earn an average of $4.00 in a performance bonus based on the decisions they make while playing the game. There will be no cost to you if you participate in this study.

Confidentiality: The data captured for the research does not include any personally identifiable information about you.

Future use of information: We may use the anonymous data for our future research studies, or we may distribute the data to other researchers for their research studies. We would do this without getting additional informed consent from you (or your legally authorized representative). Sharing of data with other researchers will only be done in such a manner that you will not be identified.

Right to ask questions & Contact Information: If you have any questions about this study, you should feel free to ask them by contacting the Principal Investigator now at Hirokazu Shirado (shirado@cmu.edu). If you have questions later, desire additional information, or wish to withdraw your participation please contact the Principal Investigator by e-mail in accordance with the contact information listed above. If you have questions pertaining to your rights as a research participant; or to report concerns to this study, you should contact the Office of Research integrity and Compliance at Carnegie Mellon University. Email: irb-review@andrew.cmu.edu . Phone: +1-412-268-1901 or +1-412-268-5460.

Voluntary participation: Your participation in this research is voluntary. You may discontinue participation at any time during the research activity. You may print a copy of this consent form for your records.

I am age 18 or older.

○ No ○ Yes

I have read and understand the information above.

○ No ○ Yes

I want to participate in this research and continue with the game.

○ No ○ Yes

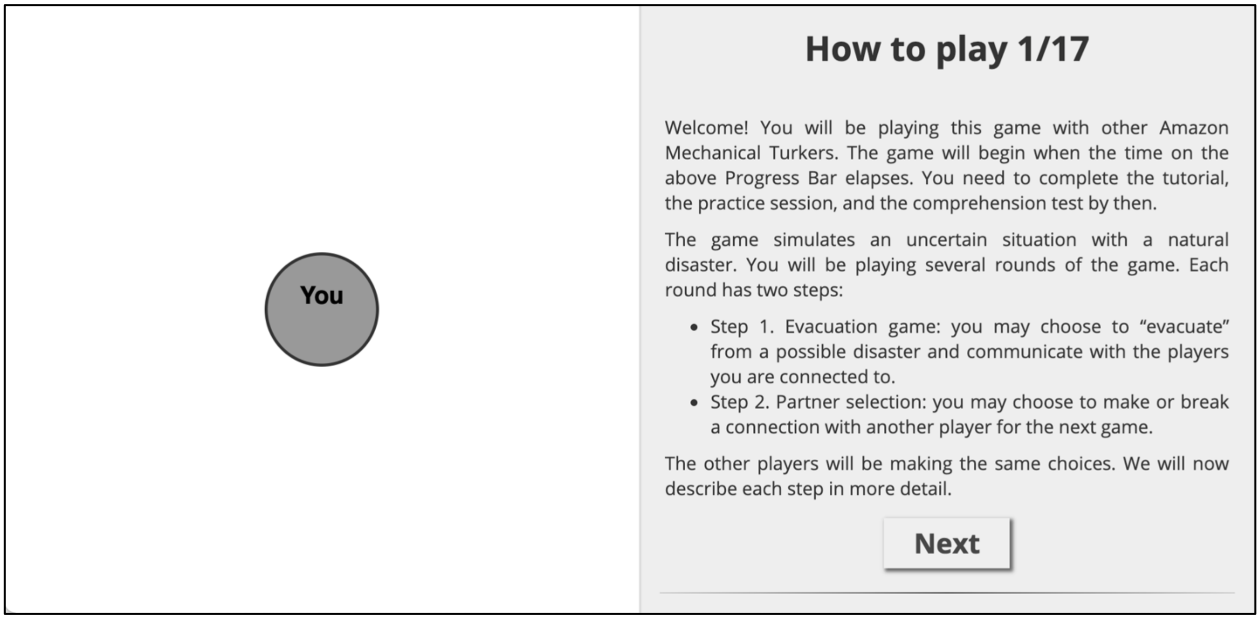
You
How to play 1/17
Welcome! You will be playing this game with other Amazon Mechanical Turkers. The game will begin when the time on the above Progress Bar elapses. You need to complete the tutorial, the practice session, and the comprehension test by then.
The game simulates an uncertain situation with a natural disaster. You will be playing several rounds of the game. Each round has two steps:
• Step 1. Evacuation game: you may choose to “evacuate” from a possible disaster and communicate with the players you are connected to.
• Step 2. Partner selection: you may choose to make or break a connection with another player for the next game.
The other players will be making the same choices. We will now describe each step in more detail.
Next

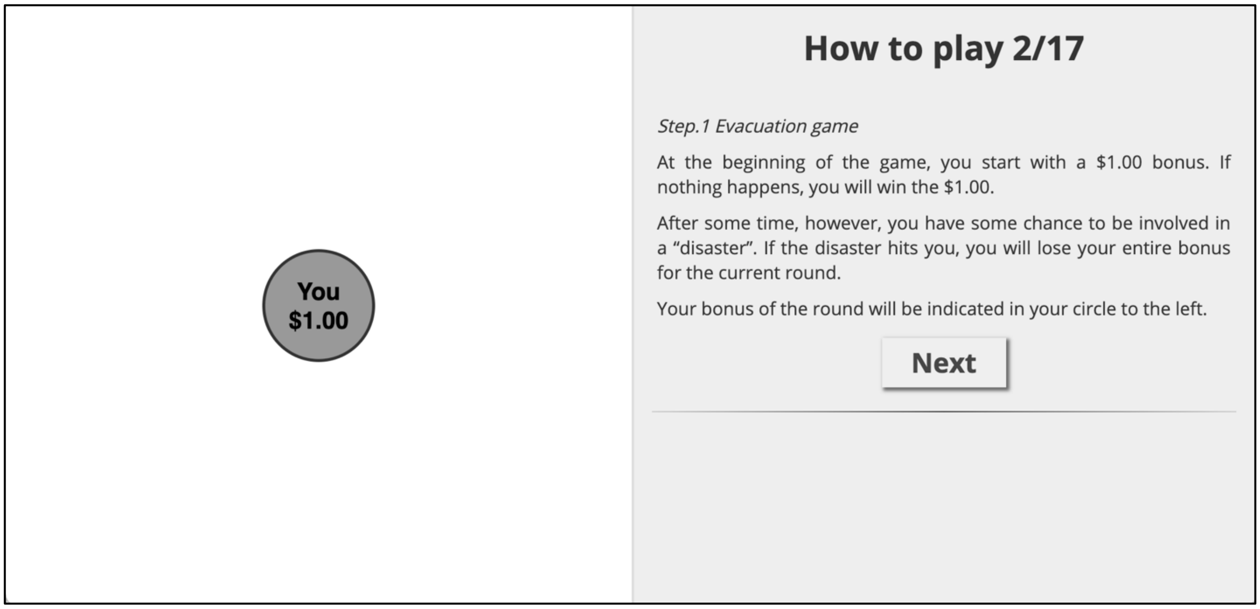
You
$1.00
How to play 2/17
Step.1 Evacuation game
At the beginning of the game, you start with a $1.00 bonus. If nothing happens, you will win the $1.00.
After some time, however, you have some chance to be involved in a “disaster”. If the disaster hits you, you will lose your entire bonus for the current round.
Your bonus of the round will be indicated in your circle to the left.
Next

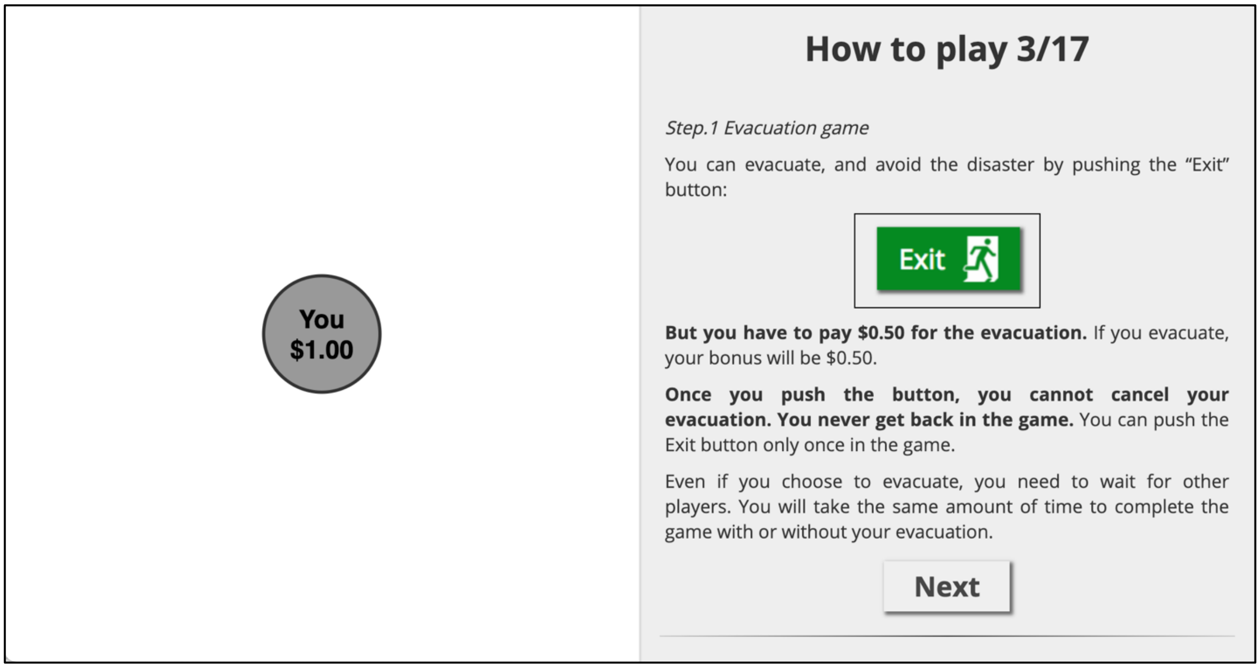
You
$1.00
How to play 3/17
Step.1 Evacuation game
You can evacuate, and avoid the disaster by pushing the "Exit" button:
Exit
But you have to pay $0.50 for the evacuation. If you evacuate, your bonus will be $0.50.
Once you push the button, you cannot cancel your evacuation. You never get back in the game. You can push the Exit button only once in the game.
Even if you choose to evacuate, you need to wait for other players. You will take the same amount of time to complete the game with or without your evacuation.
Next

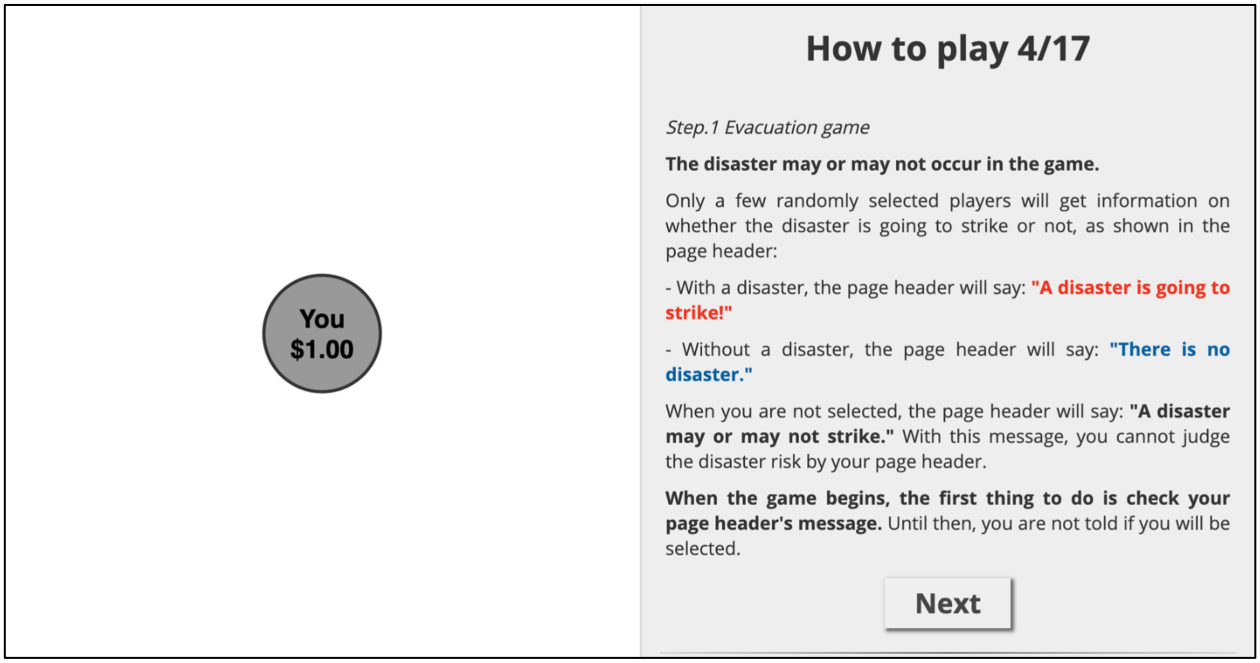
You
$1.00
How to play 4/17
Step.1 Evacuation game
The disaster may or may not occur in the game.
Only a few randomly selected players will get information on whether the disaster is going to strike or not, as shown in the page header:
- With a disaster, the page header will say: "A disaster is going to strike!"
- Without a disaster, the page header will say: "There is no disaster."
When you are not selected, the page header will say: "A disaster may or may not strike." With this message, you cannot judge the disaster risk by your page header.
When the game begins, the first thing to do is check your page header's message. Until then, you are not told if you will be selected.
Next

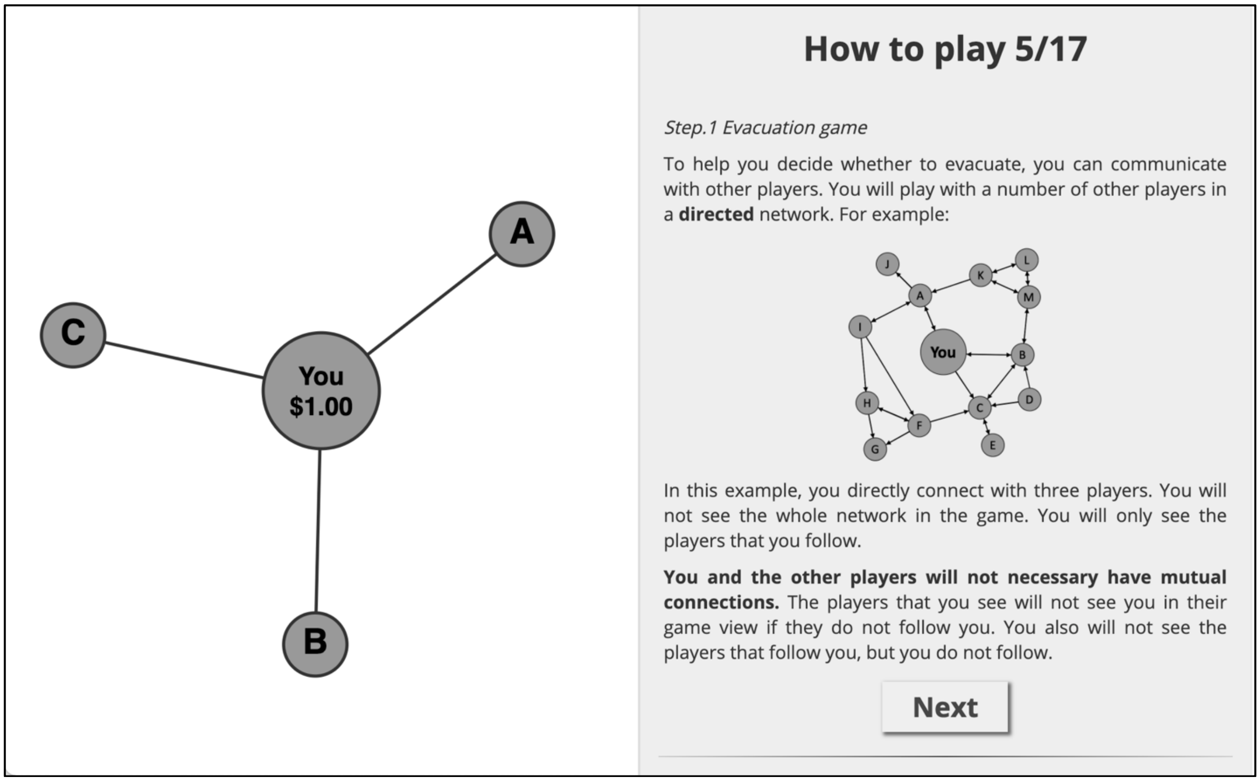
A
C
You
$1.00
B
How to play 5/17
Step.1 Evacuation game
To help you decide whether to evacuate, you can communicate with other players. You will play with a number of other players in a directed network. For example:
J
K
L
A
M
I
You
B
H
C
D
F
G
E
In this example, you directly connect with three players. You will not see the whole network in the game. You will only see the players that you follow.
You and the other players will not necessary have mutual connections. The players that you see will not see you in their game view if they do not follow you. You also will not see the players that follow you, but you do not follow.
Next

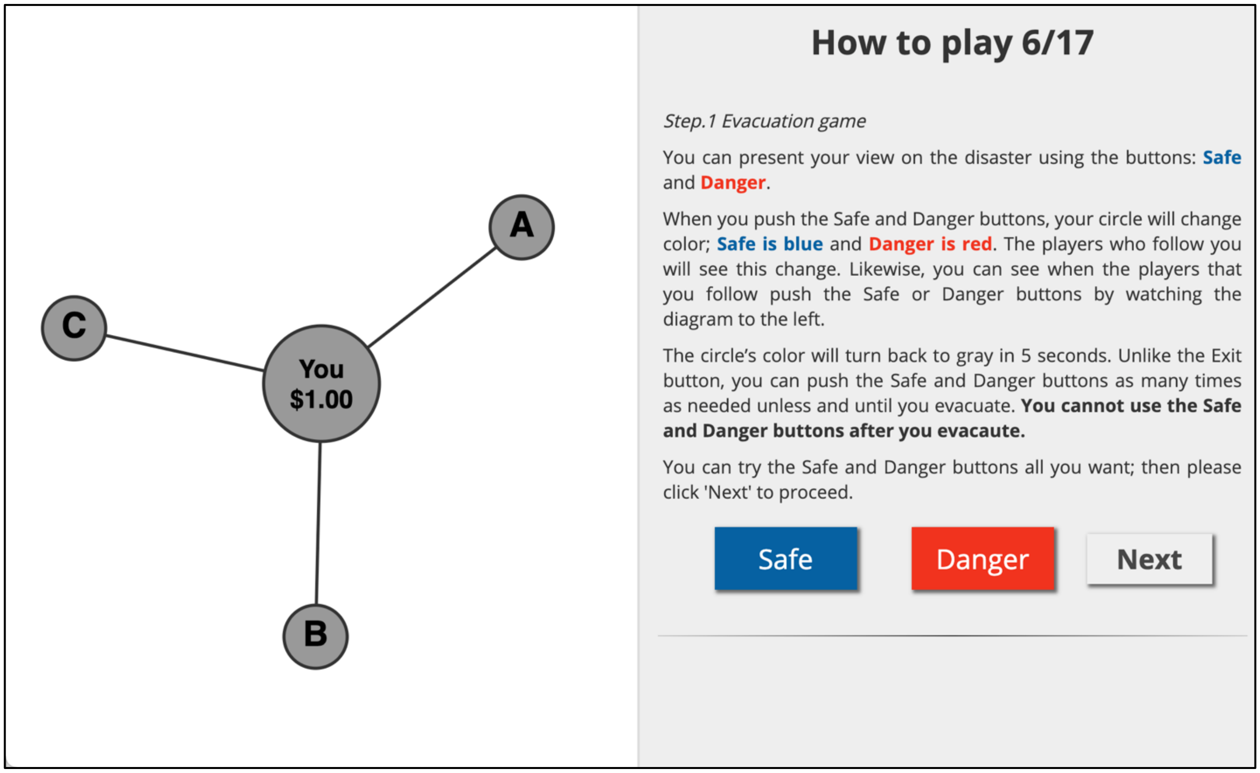
A
C
You
$1.00
B
How to play 6/17
Step.1 Evacuation game
You can present your view on the disaster using the buttons: Safe and Danger.
When you push the Safe and Danger buttons, your circle will change color; Safe is blue and Danger is red. The players who follow you will see this change. Likewise, you can see when the players that you follow push the Safe or Danger buttons by watching the diagram to the left.
The circle's color will turn back to gray in 5 seconds. Unlike the Exit button, you can push the Safe and Danger buttons as many times as needed unless and until you evacuate. You cannot use the Safe and Danger buttons after you evacaute.
You can try the Safe and Danger buttons all you want; then please click 'Next' to proceed.
Safe
Danger
Next

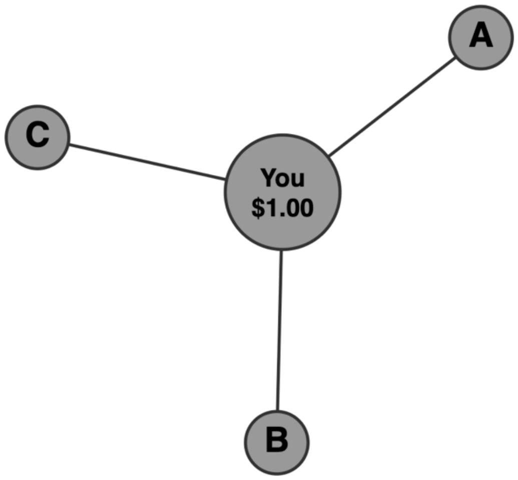

How to play 7/17

Step.1 Evacuation game

By pushing the Safe button, you can communicate to your neighbors that you think it is safe to remain in the game.

By pushing the Danger button, you can communicate to your neighbors that you think you are in danger of being involved in the disaster.

You cannot use the Safe and Danger buttons after you evacuate. Evacuated players can no longer communicate using the Safe and Danger buttons.

The network diagram doesn't change even if you or your neighbors evacuate; that is, if a neighbor evacuates, you will still be connected to a gray circle. If a circle turns blue or red, that player has not yet evacuated.

Next

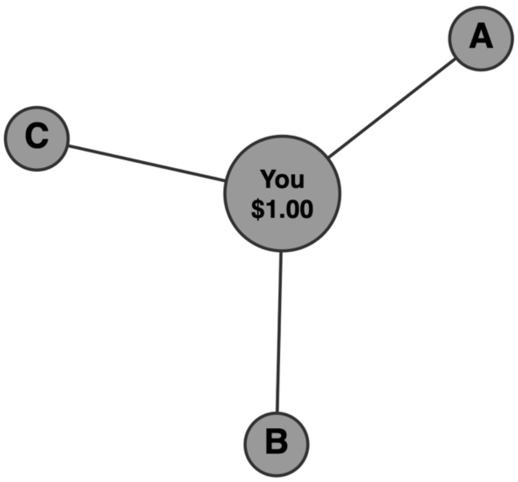

How to play 8/17

Step.1 Evacuation game

Unless you are involved in the disaster, you might earn extra bonus from other players in your group.

In addition to the default bonus, you will earn $0.05 per player who takes a correct action in the network. If you share accurate information with other players, more of them will make the correct decision, and you will get a larger amount of money added to your bonus.

As noted before, you cannot use the Safe and Danger buttons after you evacuate. You will need to communicate before you evacuate.

Next

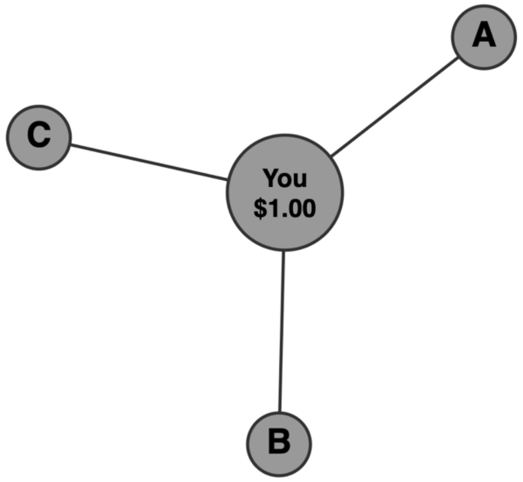

How to play 9/17

Step.1 Evacuation game

In sum, you can earn a bonus at the end of each game depending on your decision and whether or not there is a disaster:

If you don't push Exit until the end of the game, you will stay on-site.

- Without a disaster, you will earn $1.00 plus $0.05 per player who stays to the end.

- With a disaster, you will earn $0.00 and no additional bonus.

If you push Exit by the end of the game, you will evacuate.

- Without a disaster, you will earn $0.50 plus $0.05 per player who stays to the end.

- With a disaster, you will earn $0.50 plus $0.05 per player who has evacuated.

Next

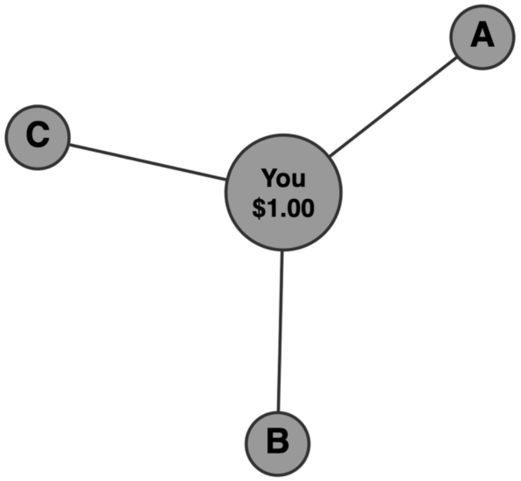

How to play 10/17

Step.1 Evacuation game

When the game ends, you will be notified whether a disaster has struck or not. If you want to evacuate, you should push Exit before this occurs.

Importantly, **all players including you are not told when the game ends.** Even if you are selected to get the disaster information, you will not get the information about when it will happen.

You may play the evacuation game just once or more in this HIT. You are not told how many rounds you are playing. After you learn of the game's result and your earnings, you may complete the HIT, or **move on to Step 2, partner selection**.

Next

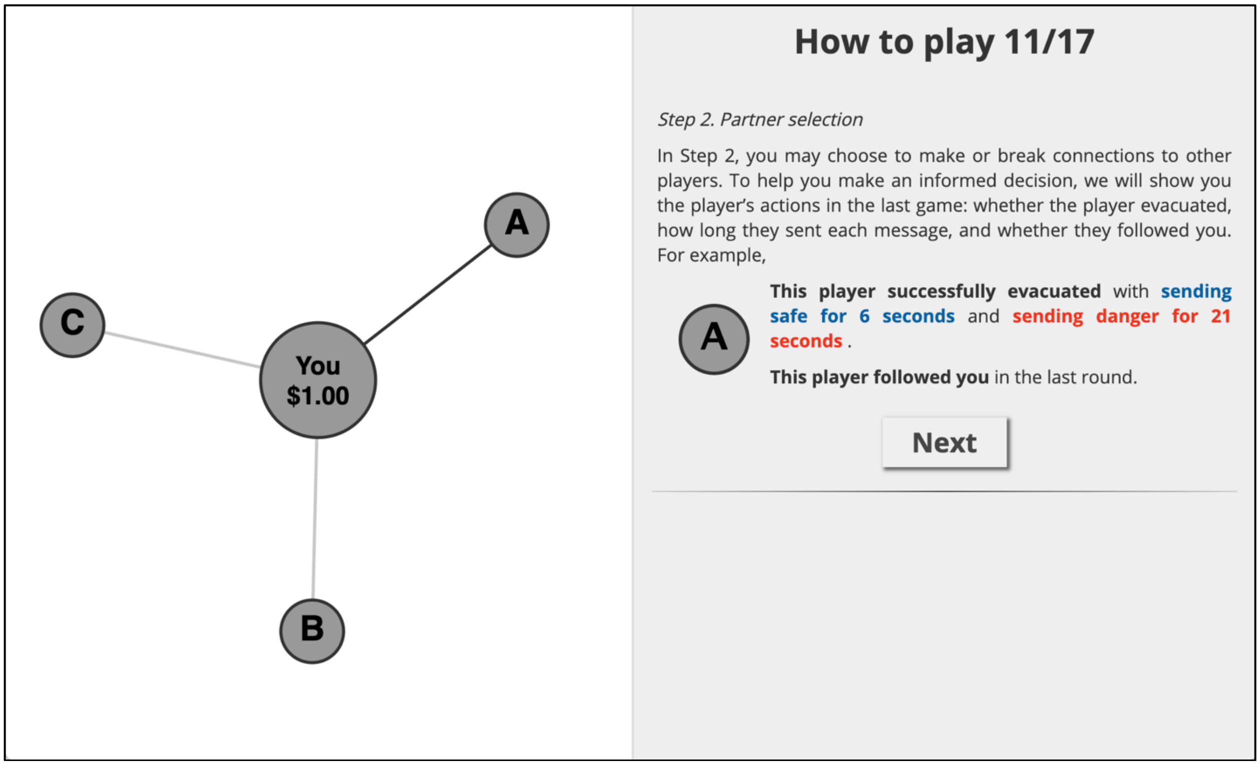
A
C
You
$1.00
B
How to play 11/17
Step 2. Partner selection
In Step 2, you may choose to make or break connections to other players. To help you make an informed decision, we will show you the player's actions in the last game: whether the player evacuated, how long they sent each message, and whether they followed you. For example,
A
This player successfully evacuated with sending safe for 6 seconds and sending danger for 21 seconds .
This player followed you in the last round.
Next

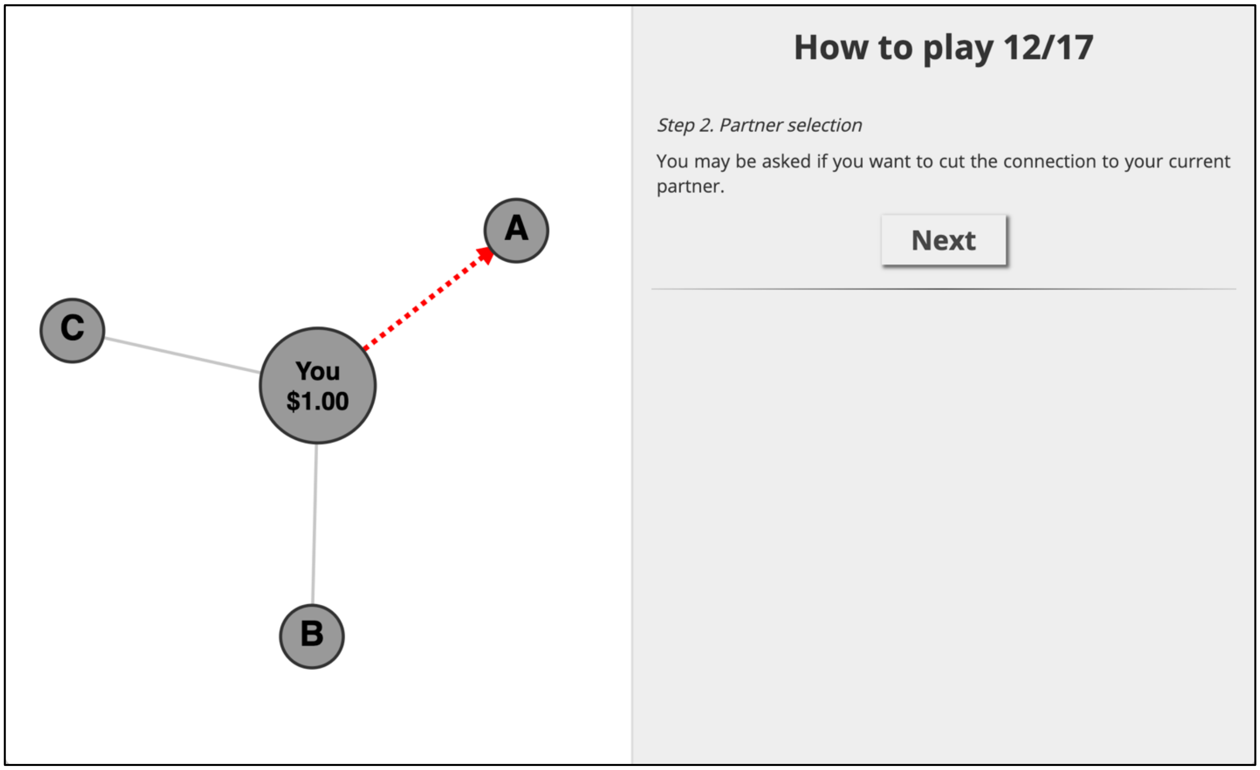
A
C
You
$1.00
B
How to play 12/17
Step 2. Partner selection
You may be asked if you want to cut the connection to your current partner.
Next

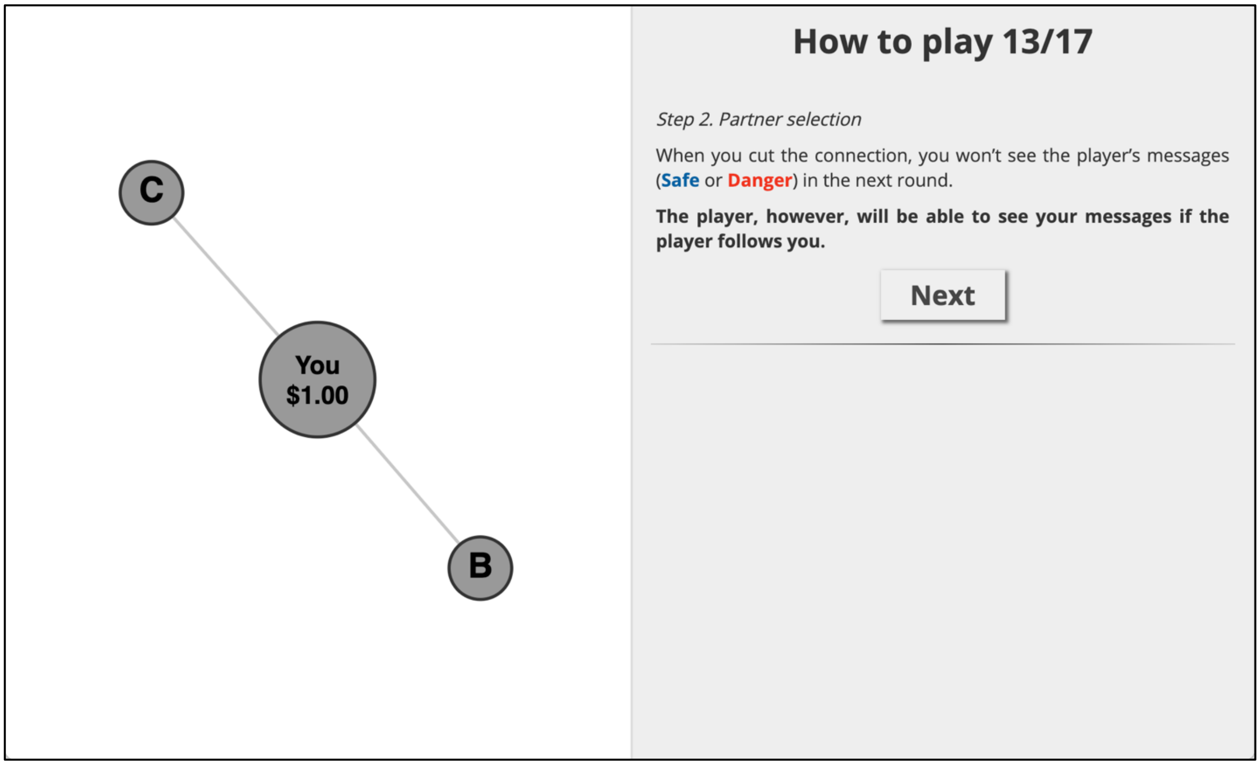
C
You
$1.00
B
How to play 13/17
Step 2. Partner selection
When you cut the connection, you won't see the player's messages (Safe or Danger) in the next round.
The player, however, will be able to see your messages if the player follows you.
Next

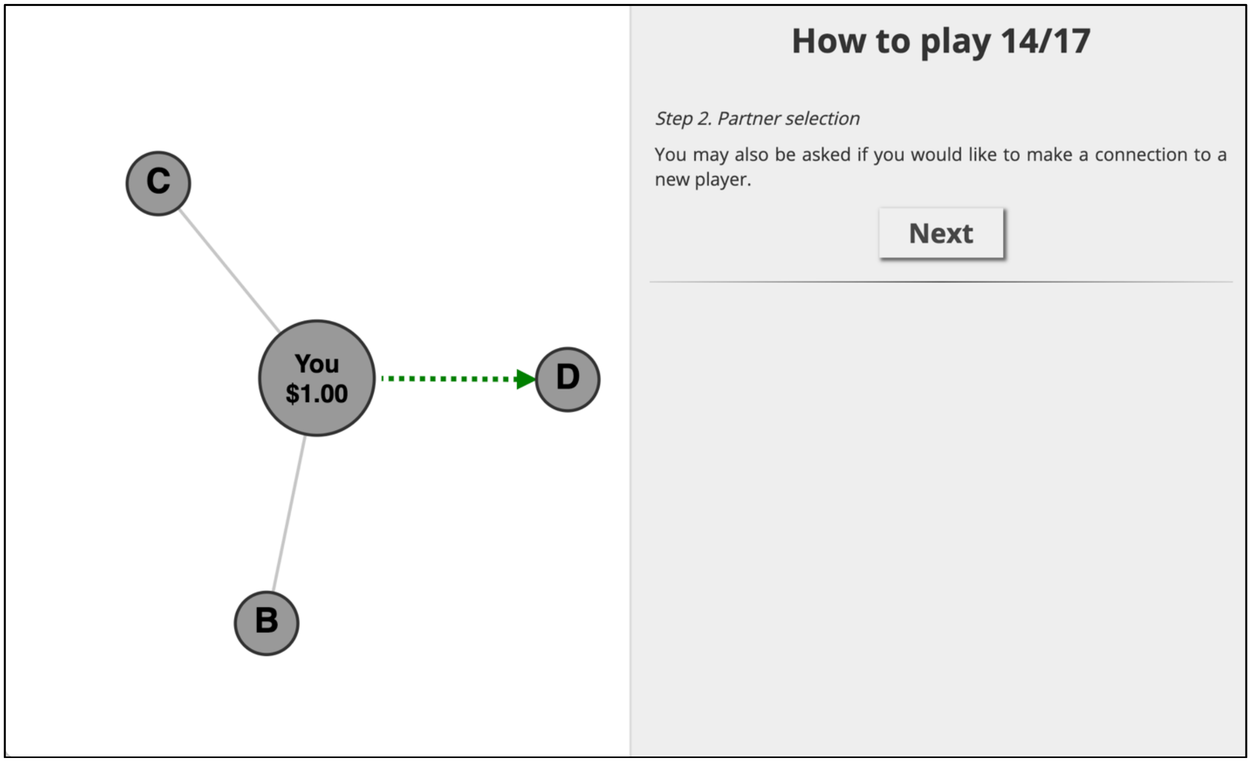
C
You
$1.00
D
B
How to play 14/17
Step 2. Partner selection
You may also be asked if you would like to make a connection to a new player.
Next

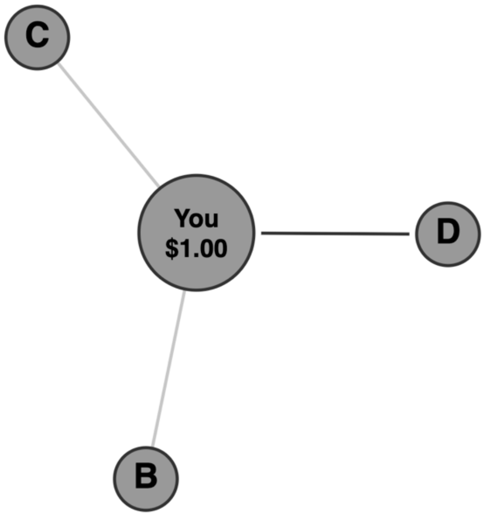

How to play 15/17

Step 2. Partner selection

When you make a connection, you will see the player's messages in the next round.

The player, however, won't see your messages if the player does not follow you.

Next

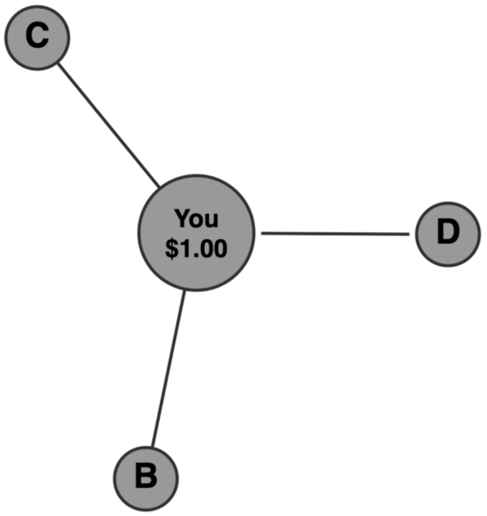

How to play 16/17

Step 2. Partner selection

If you have the next round, you will play the evacuation game again with your updated connections. Note that you will be removed from the game if other players are waiting on you to make your partner selections for longer than 20 seconds.

When you participate in all the round(s) and complete the game, you will be paid $1.00 as completion bonus in addition to your total earnings in the game.

Next

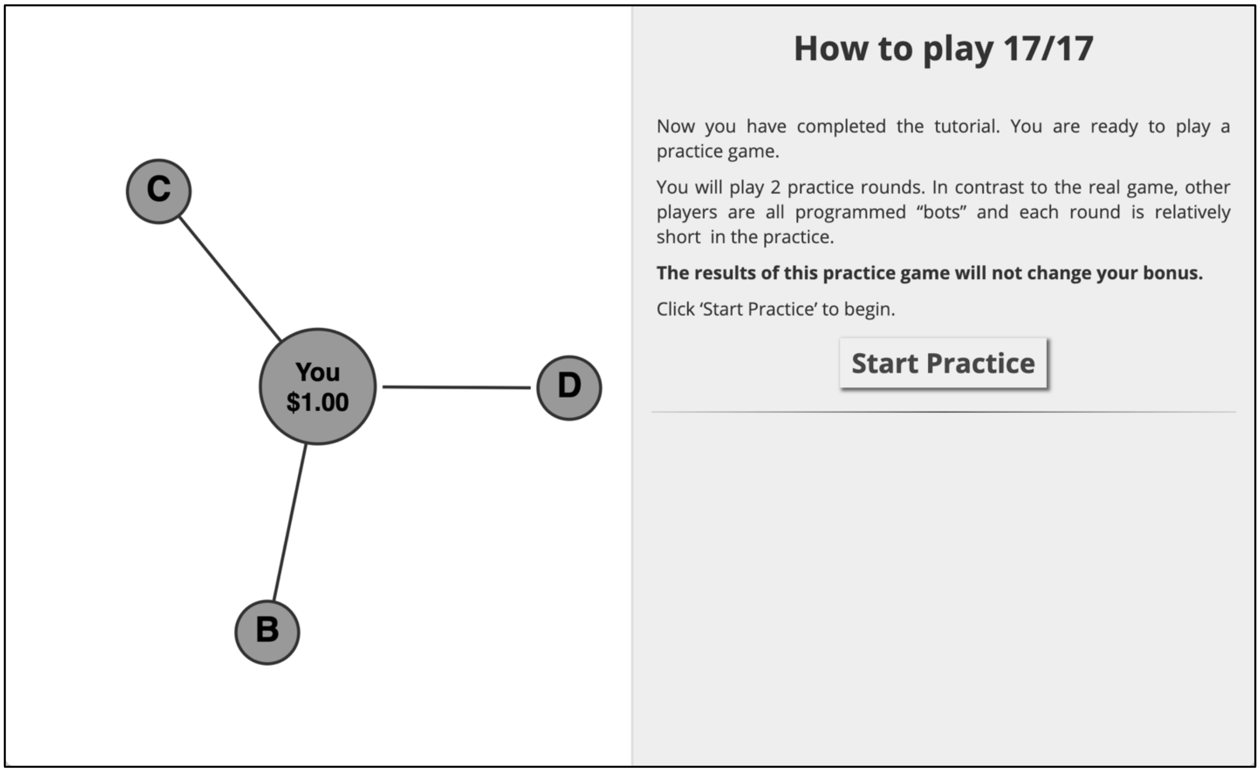
C
You
$1.00
D
B
How to play 17/17
Now you have completed the tutorial. You are ready to play a practice game.
You will play 2 practice rounds. In contrast to the real game, other players are all programmed “bots” and each round is relatively short in the practice.
The results of this practice game will not change your bonus.
Click ‘Start Practice’ to begin.
Start Practice

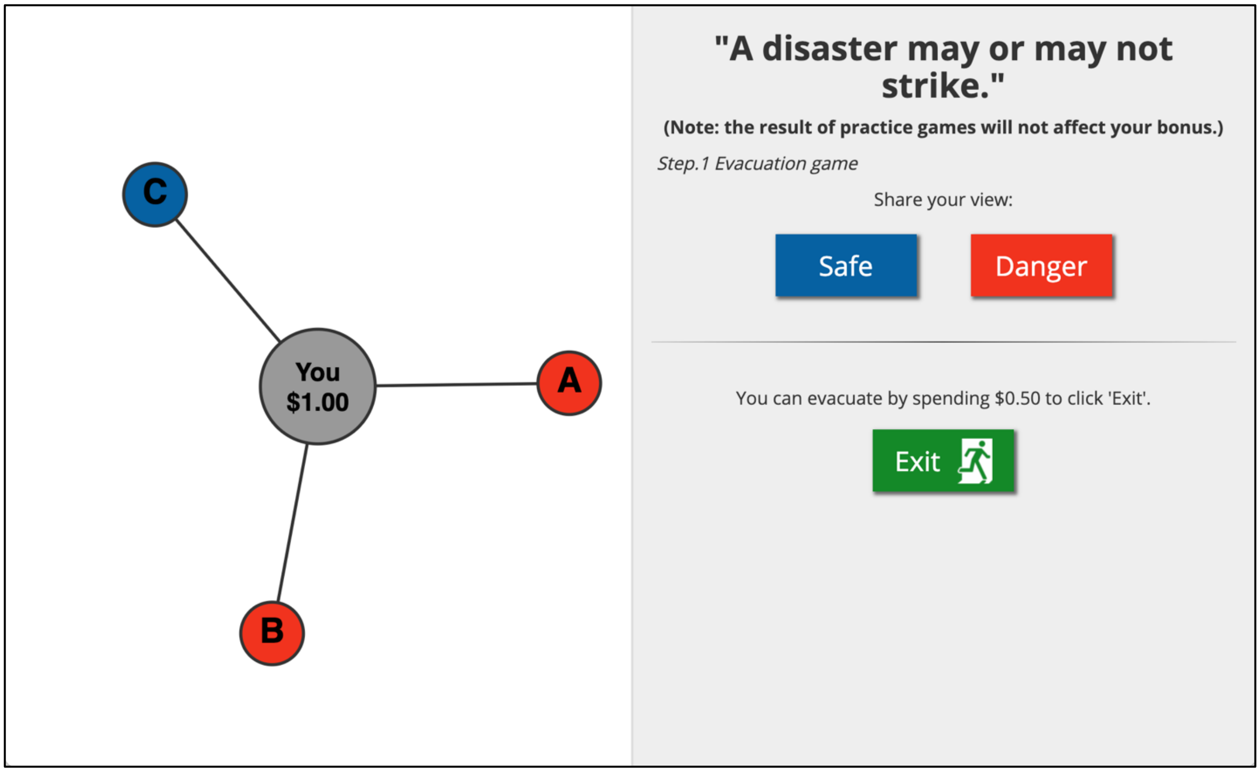
C
You
$1.00
A
B
"A disaster may or may not strike."
(Note: the result of practice games will not affect your bonus.)
Step.1 Evacuation game
Share your view:
Safe
Danger
You can evacuate by spending $0.50 to click 'Exit'.
Exit

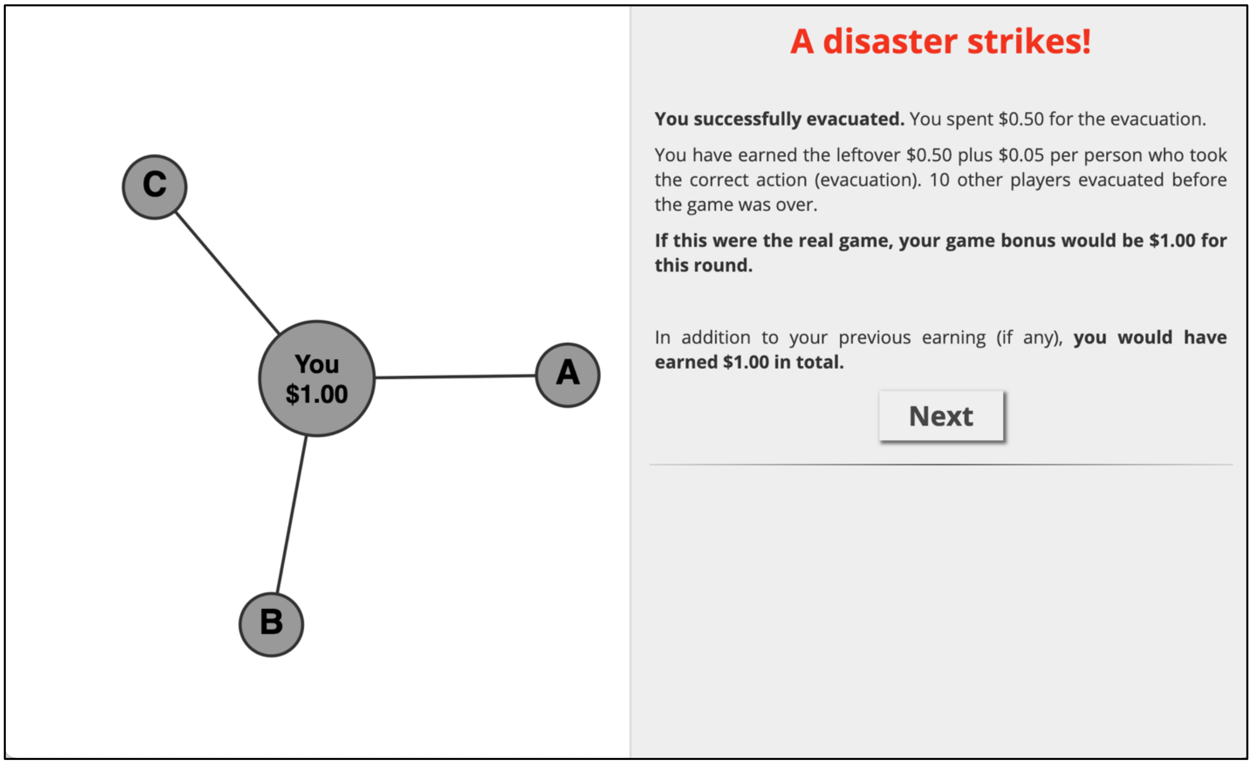
C
You
$1.00
A
B
A disaster strikes!
You successfully evacuated. You spent $0.50 for the evacuation.
You have earned the leftover $0.50 plus $0.05 per person who took the correct action (evacuation). 10 other players evacuated before the game was over.
If this were the real game, your game bonus would be $1.00 for this round.
In addition to your previous earning (if any), you would have earned $1.00 in total.
Next

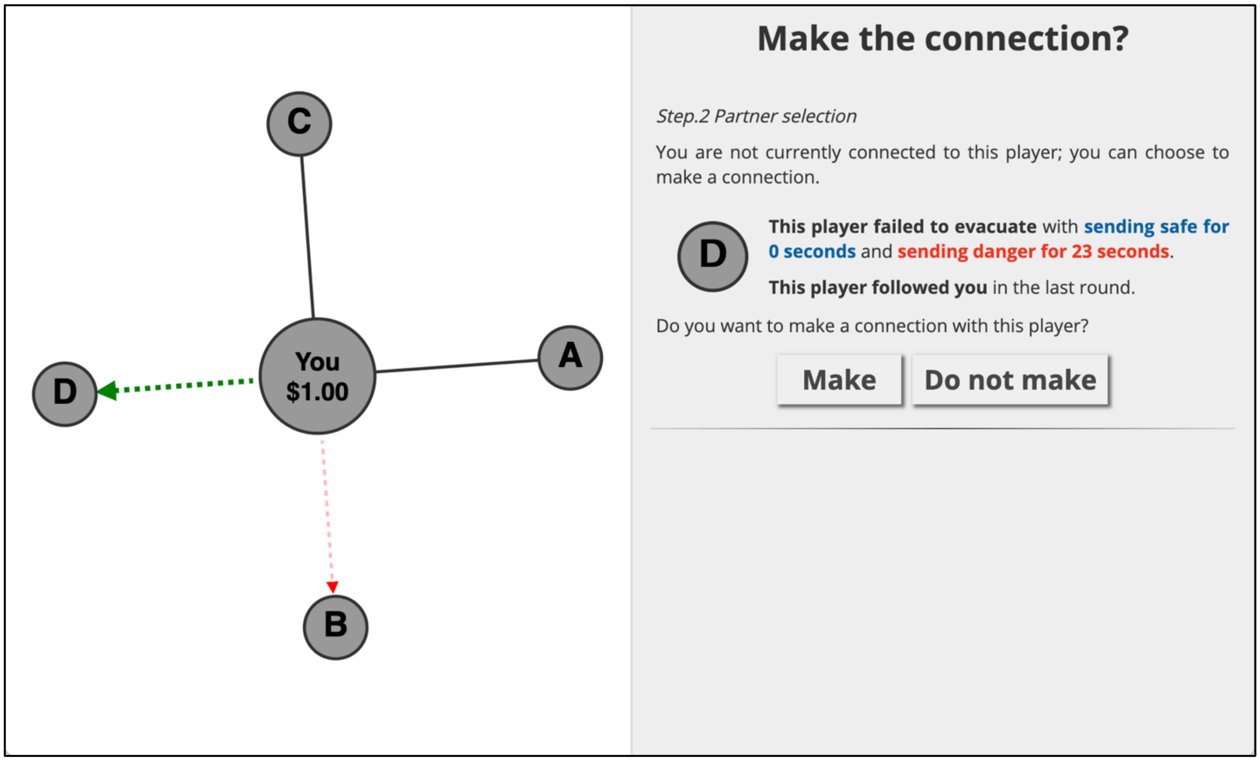
C
D
You
$1.00
A
B
Make the connection?
Step.2 Partner selection
You are not currently connected to this player; you can choose to make a connection.
D
This player failed to evacuate with sending safe for 0 seconds and sending danger for 23 seconds.
This player followed you in the last round.
Do you want to make a connection with this player?
Make
Do not make

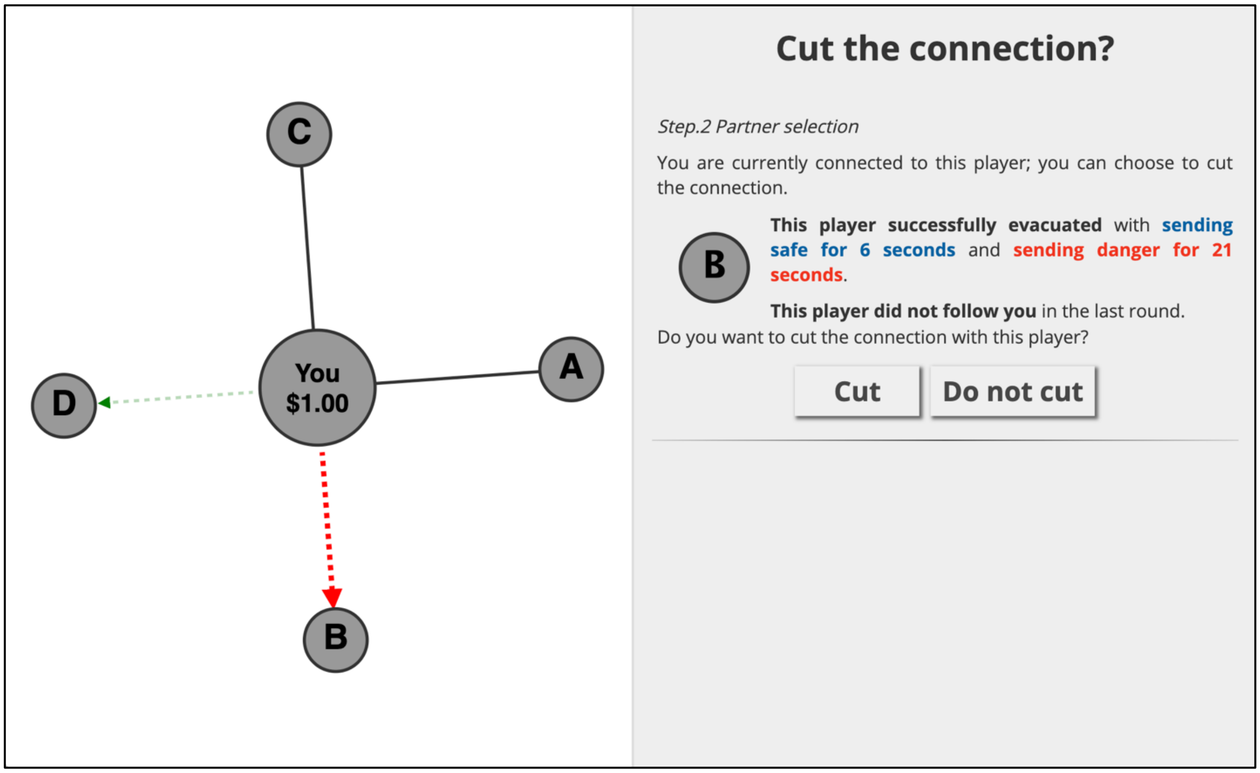
C
You
$1.00
D
A
B
Cut the connection?
Step.2 Partner selection
You are currently connected to this player; you can choose to cut the connection.
B
This player successfully evacuated with sending safe for 6 seconds and sending danger for 21 seconds.
This player did not follow you in the last round.
Do you want to cut the connection with this player?
Cut
Do not cut

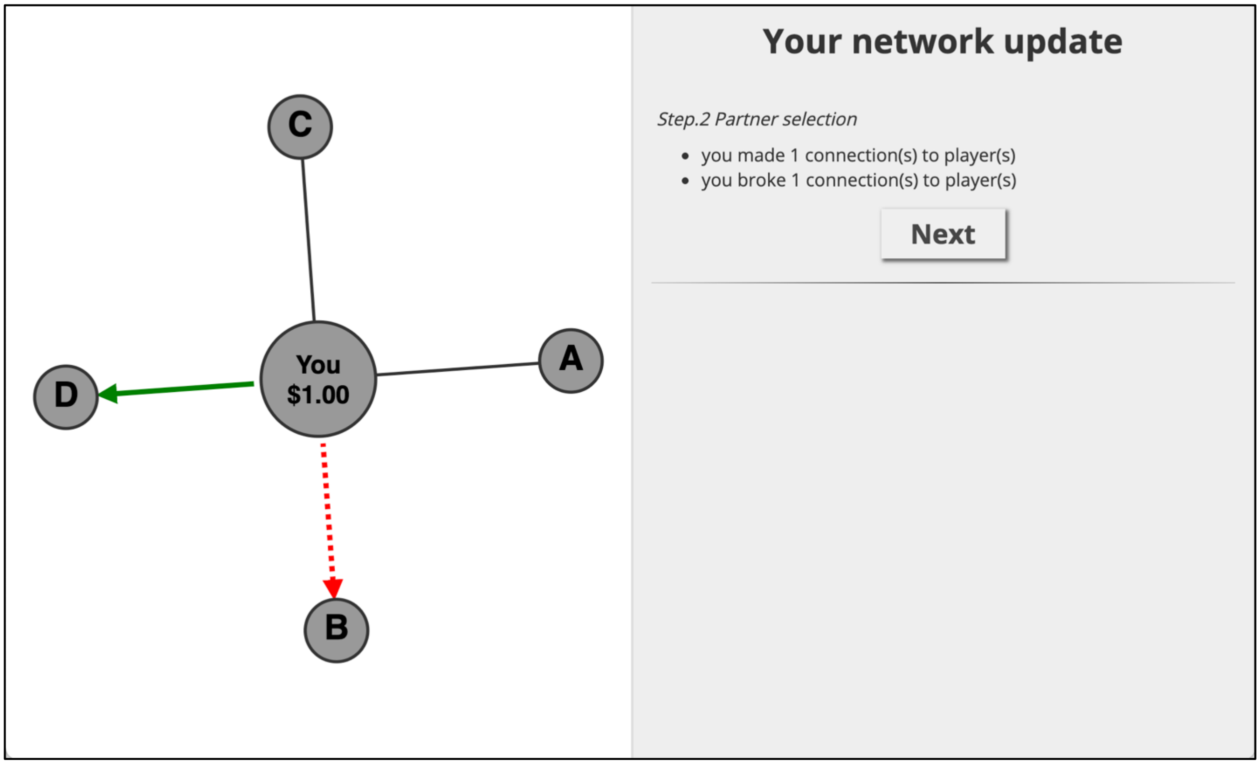
C
You
$1.00
D
A
B
Your network update
Step.2 Partner selection
you made 1 connection(s) to player(s)
you broke 1 connection(s) to player(s)
Next

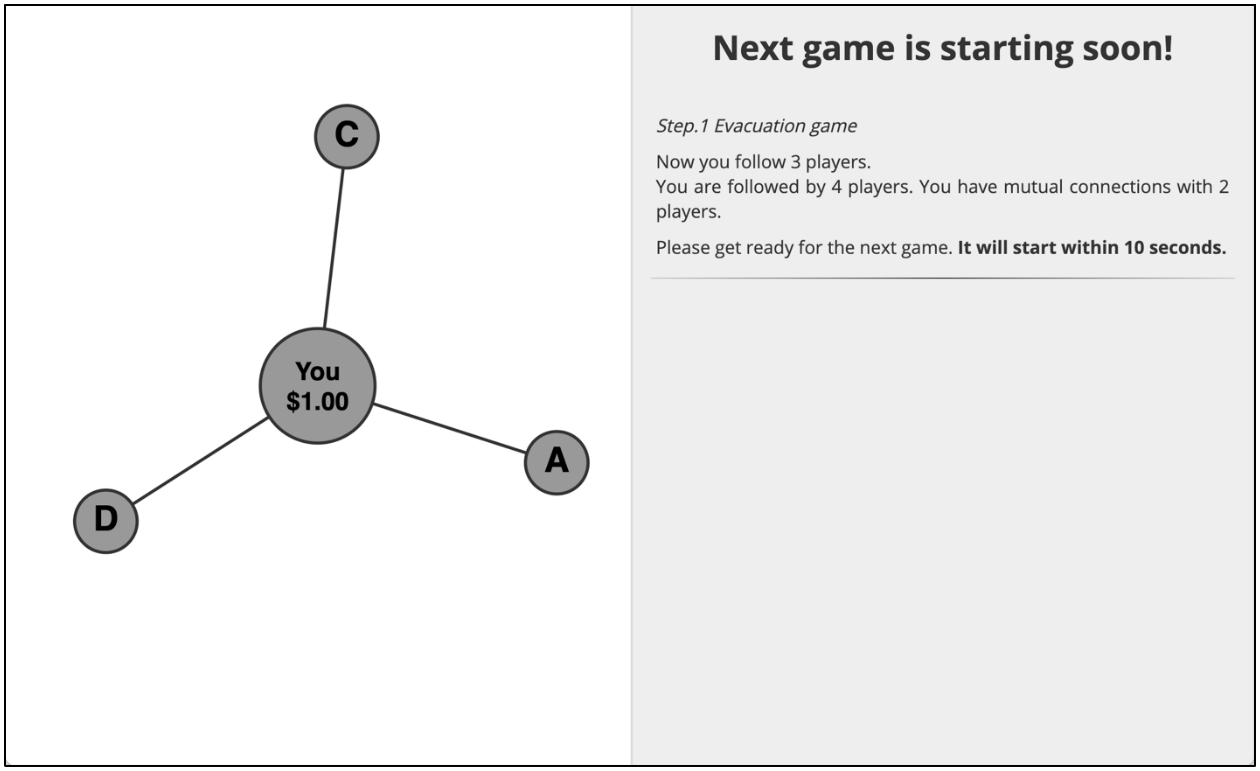
C
You
$1.00
A
D
Next game is starting soon!
Step.1 Evacuation game
Now you follow 3 players.
You are followed by 4 players. You have mutual connections with 2 players.
Please get ready for the next game. It will start within 10 seconds.

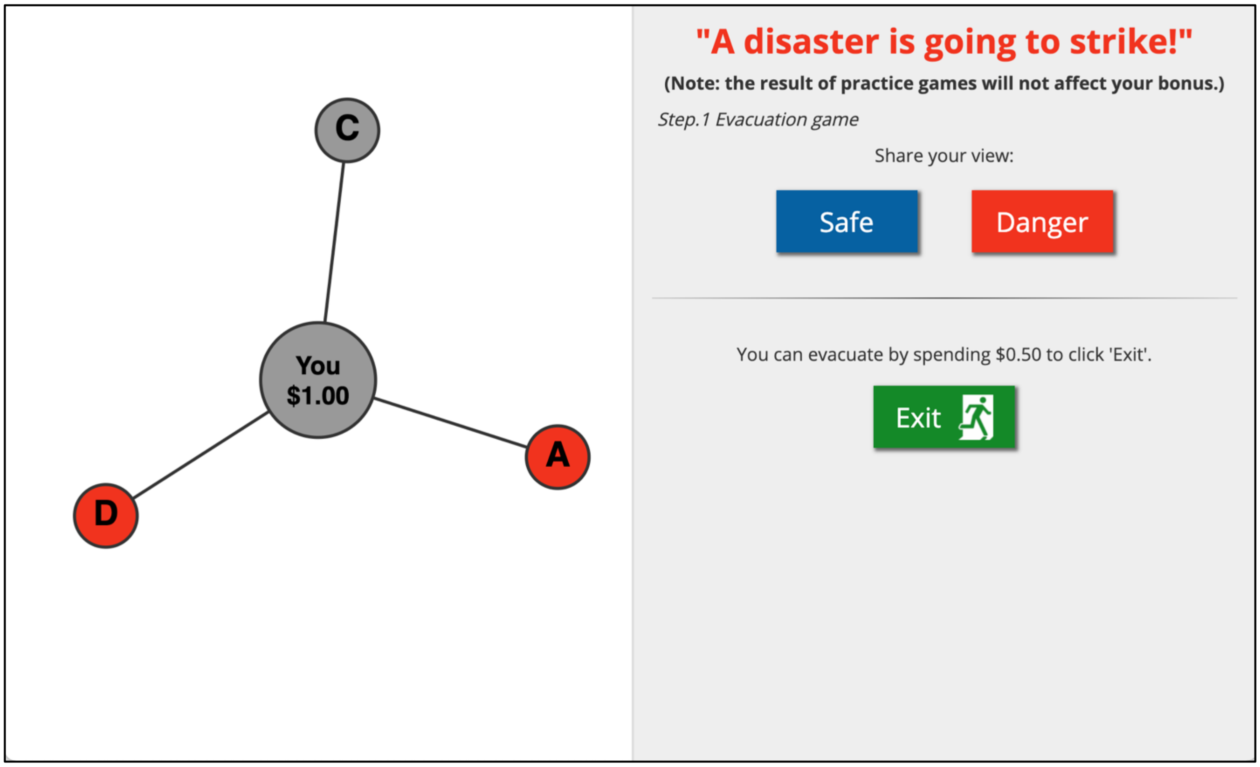
C
You
$1.00
A
D
"A disaster is going to strike!"
(Note: the result of practice games will not affect your bonus.)
Step.1 Evacuation game
Share your view:
Safe
Danger
You can evacuate by spending $0.50 to click 'Exit'.
Exit

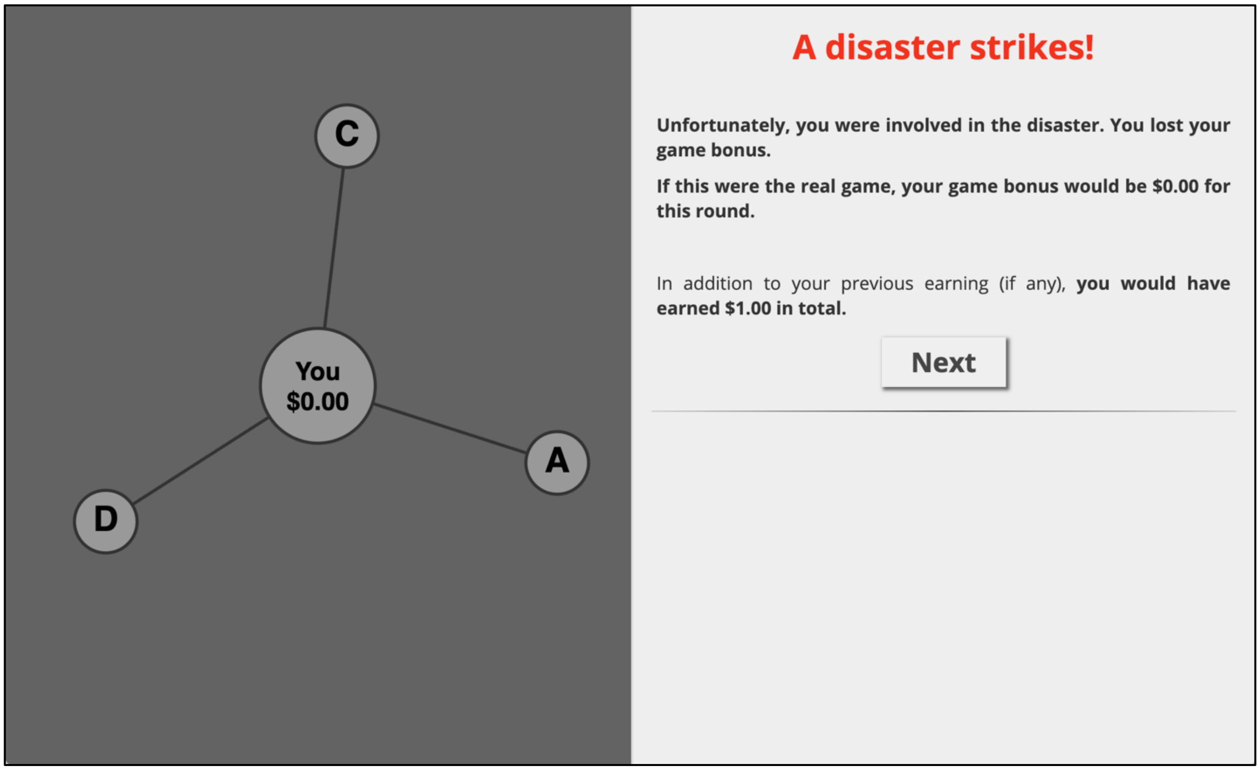
C
You
$0.00
A
D
A disaster strikes!
Unfortunately, you were involved in the disaster. You lost your game bonus.
If this were the real game, your game bonus would be $0.00 for this round.
In addition to your previous earning (if any), you would have earned $1.00 in total.
Next

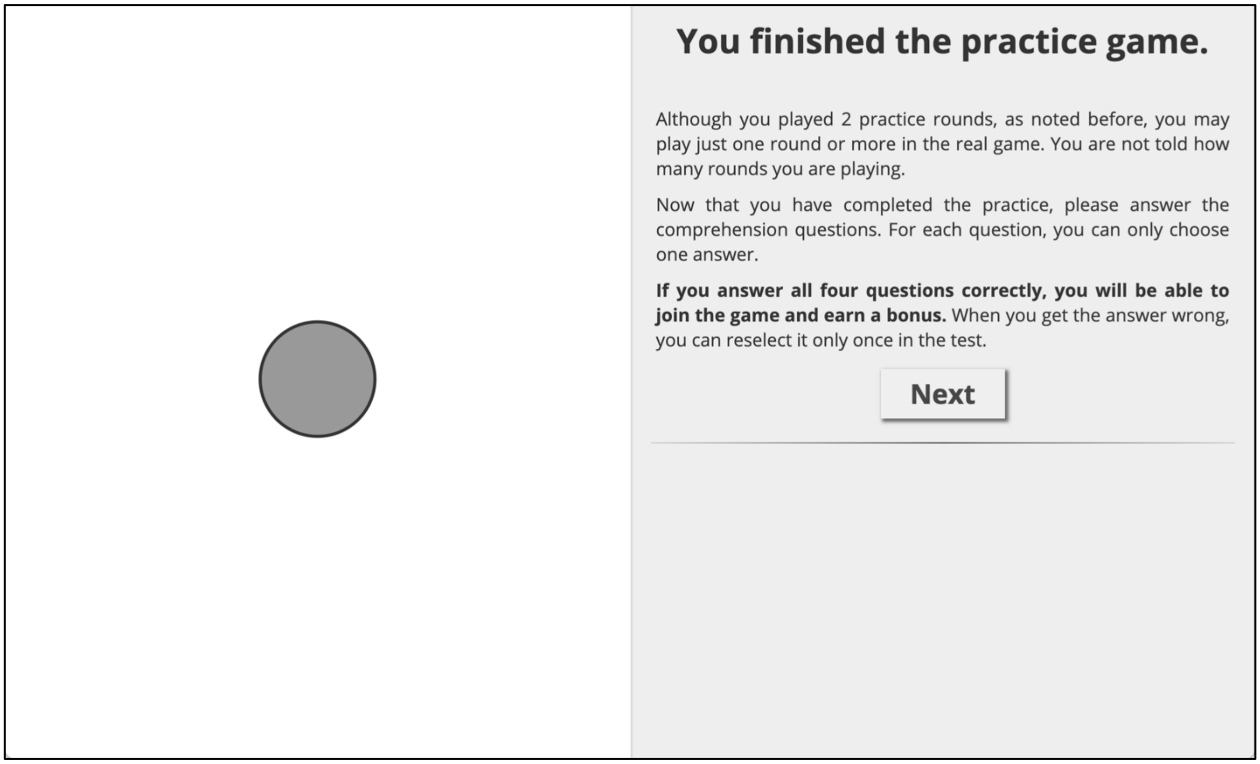
You finished the practice game.
Although you played 2 practice rounds, as noted before, you may play just one round or more in the real game. You are not told how many rounds you are playing.
Now that you have completed the practice, please answer the comprehension questions. For each question, you can only choose one answer.
If you answer all four questions correctly, you will be able to join the game and earn a bonus. When you get the answer wrong, you can reselect it only once in the test.
Next

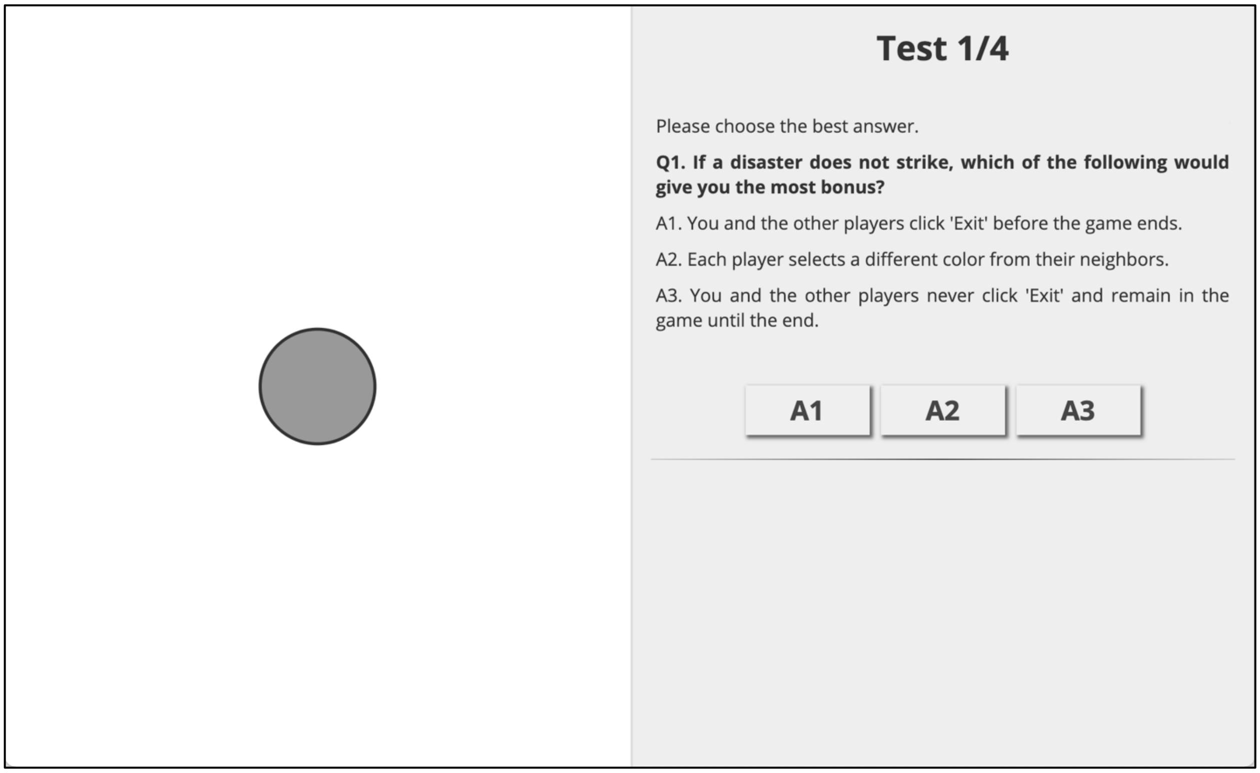
Test 1/4
Please choose the best answer.
Q1. If a disaster does not strike, which of the following would give you the most bonus?
A1. You and the other players click 'Exit' before the game ends.
A2. Each player selects a different color from their neighbors.
A3. You and the other players never click 'Exit' and remain in the game until the end.
A1
A2
A3

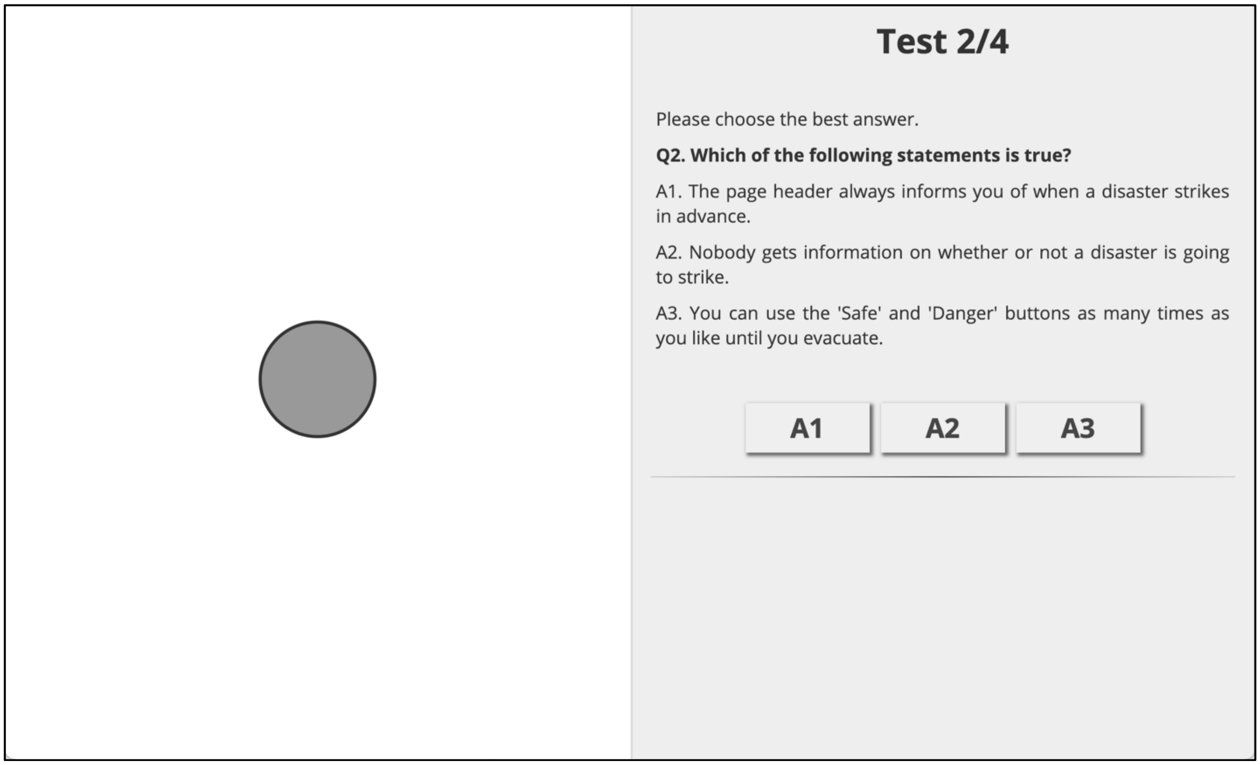
Test 2/4
Please choose the best answer.
Q2. Which of the following statements is true?
A1. The page header always informs you of when a disaster strikes in advance.
A2. Nobody gets information on whether or not a disaster is going to strike.
A3. You can use the 'Safe' and 'Danger' buttons as many times as you like until you evacuate.
A1
A2
A3

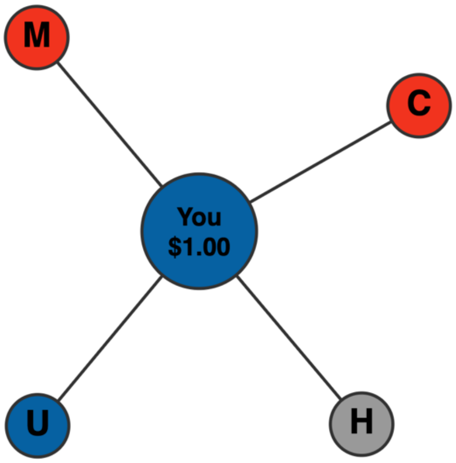

Test 3/4

Please choose the best answer.

Q3. Which sentence properly explains the situation to the left?

A1. Two of your neighbors clicked the 'Danger' button during the last 5 seconds.

A2. There are only four players in the entire network.

A3. You should change your circle color to green.

A1 A2 A3

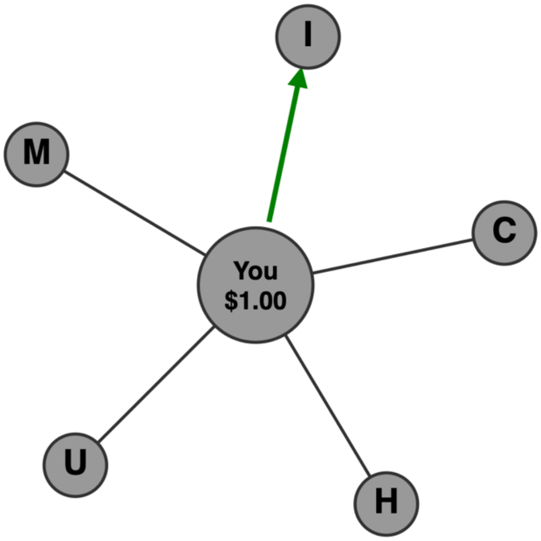

Test 4/4

Please choose the best answer.

Q4. You chose to make a connection to the new player. What happens next?

A1. This situation would not occur; you will play with the same neighbors every round.

A2. You will exchange greetings with the player before the next game begins.

A3. You will follow the player and see the player's messages in the next game.

A1 A2 A3

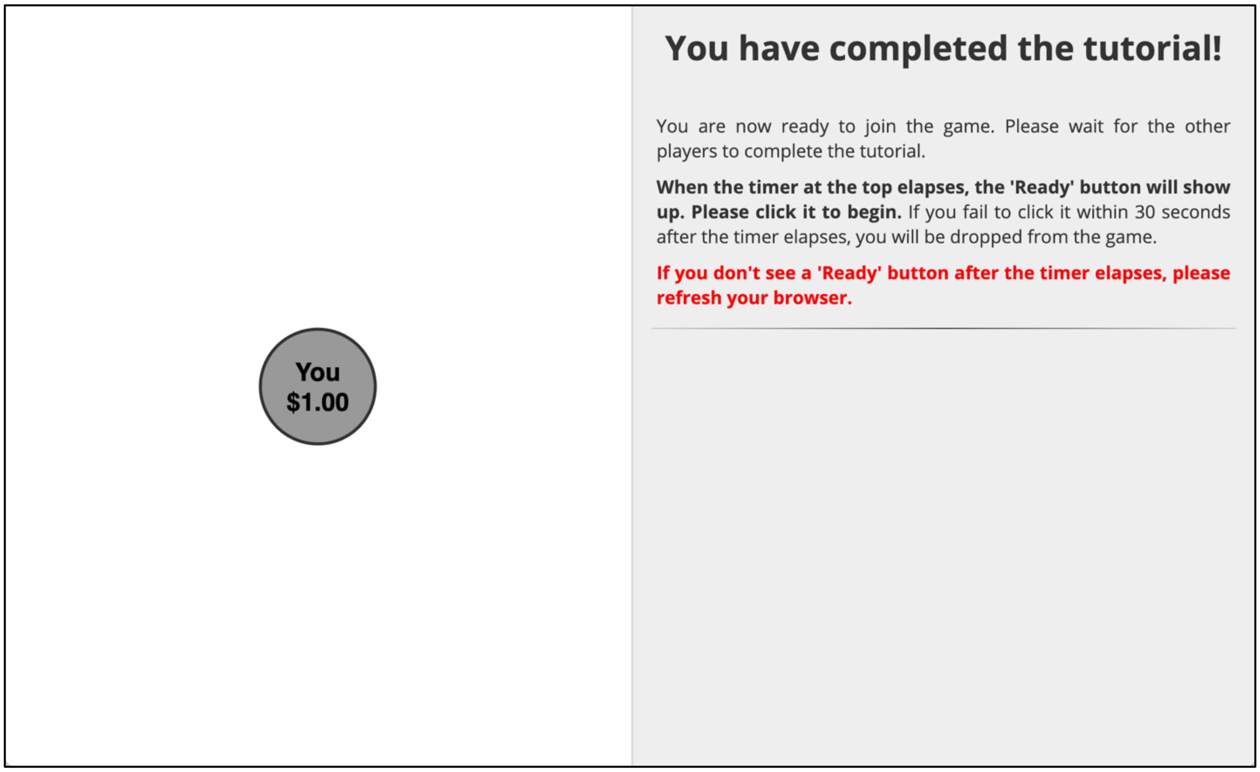

The following screenshots are the samples of the first two rounds of an actual game in the dynamic network condition.

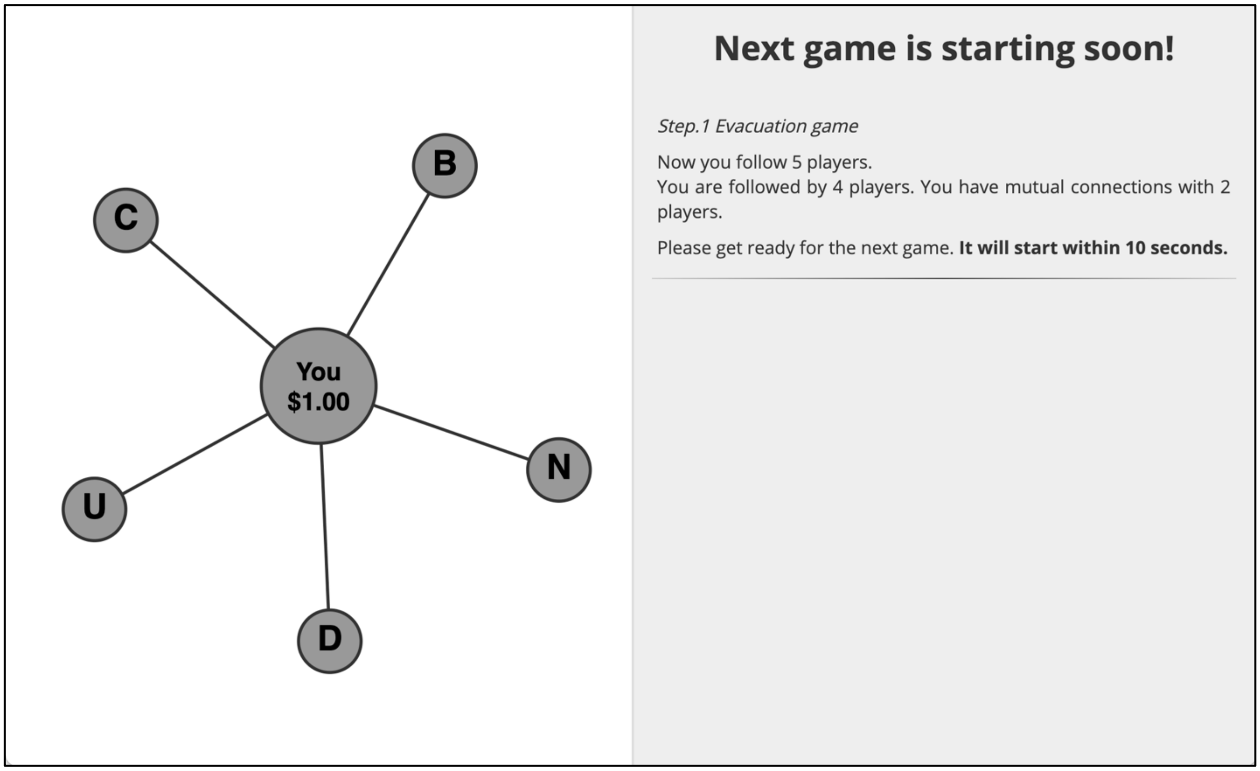

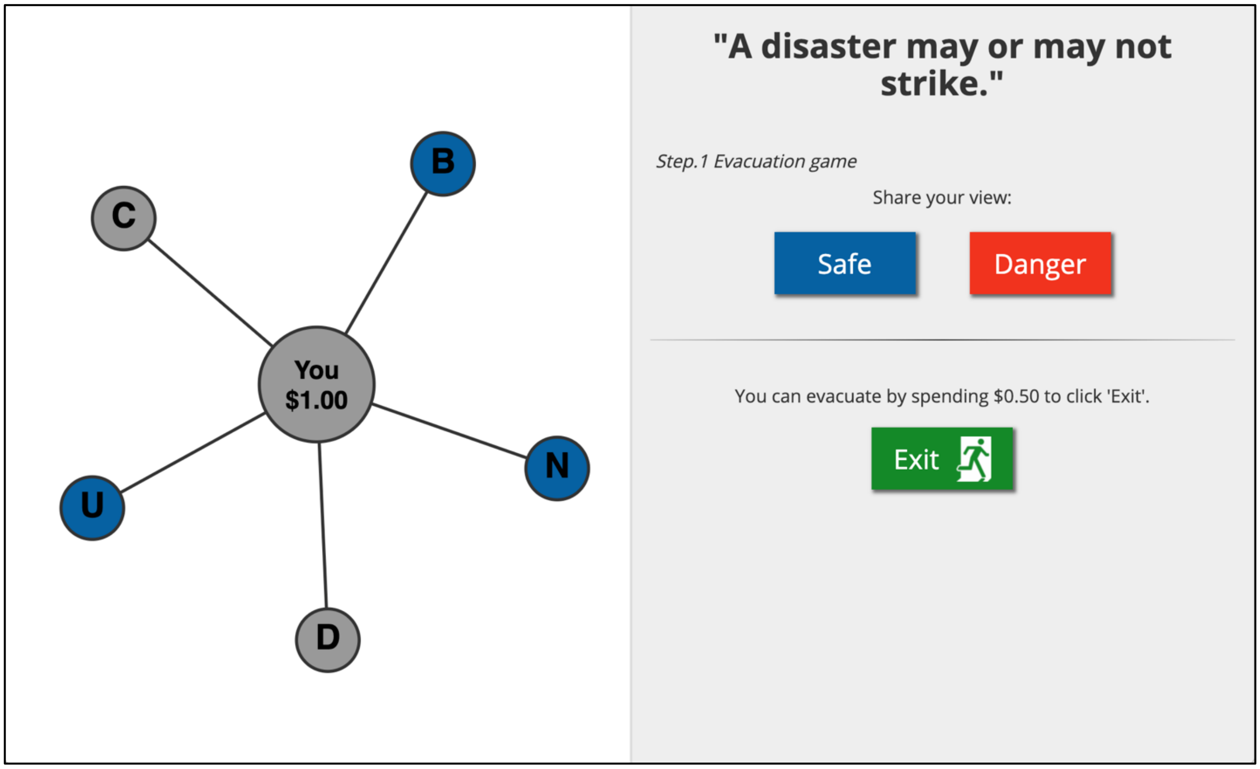
C
B
You
$1.00
U
N
D
"A disaster may or may not strike."
Step.1 Evacuation game
Share your view:
Safe
Danger
You can evacuate by spending $0.50 to click 'Exit'.
Exit

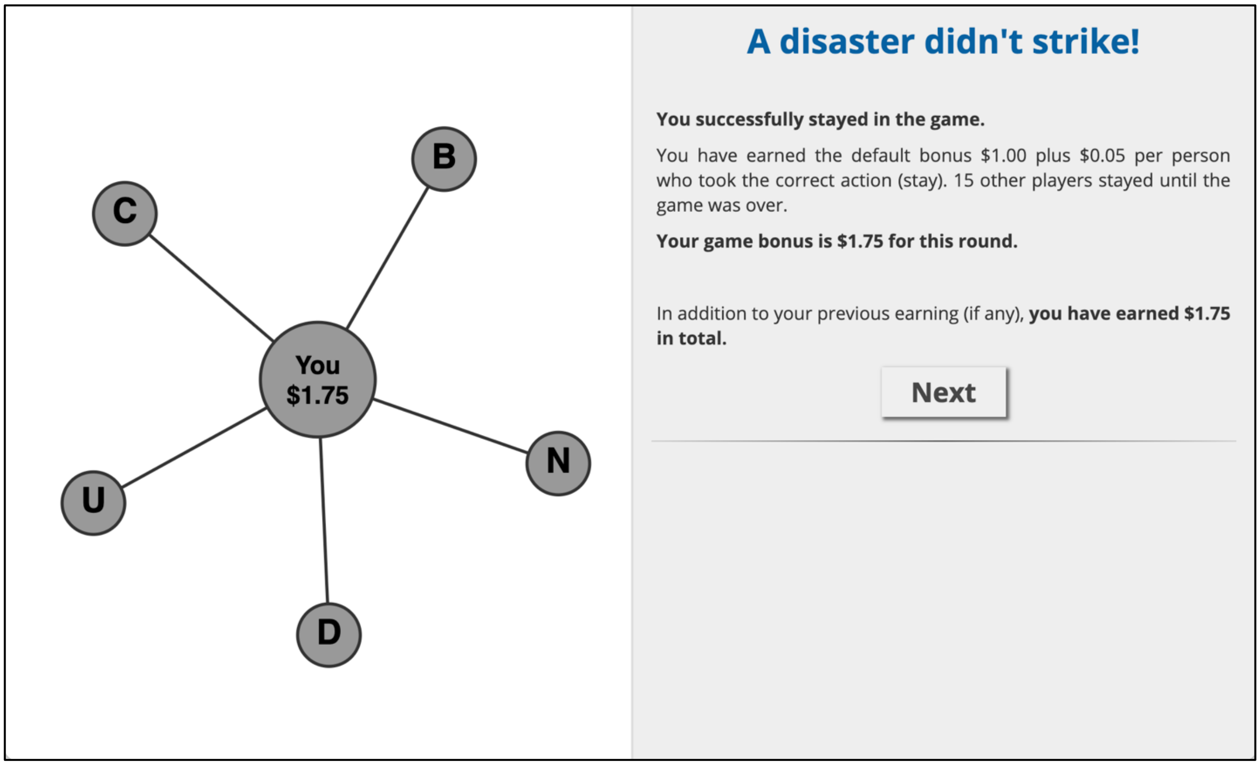
B
C
You
$1.75
U
N
D
A disaster didn't strike!
You successfully stayed in the game.
You have earned the default bonus $1.00 plus $0.05 per person who took the correct action (stay). 15 other players stayed until the game was over.
Your game bonus is $1.75 for this round.
In addition to your previous earning (if any), you have earned $1.75 in total.
Next

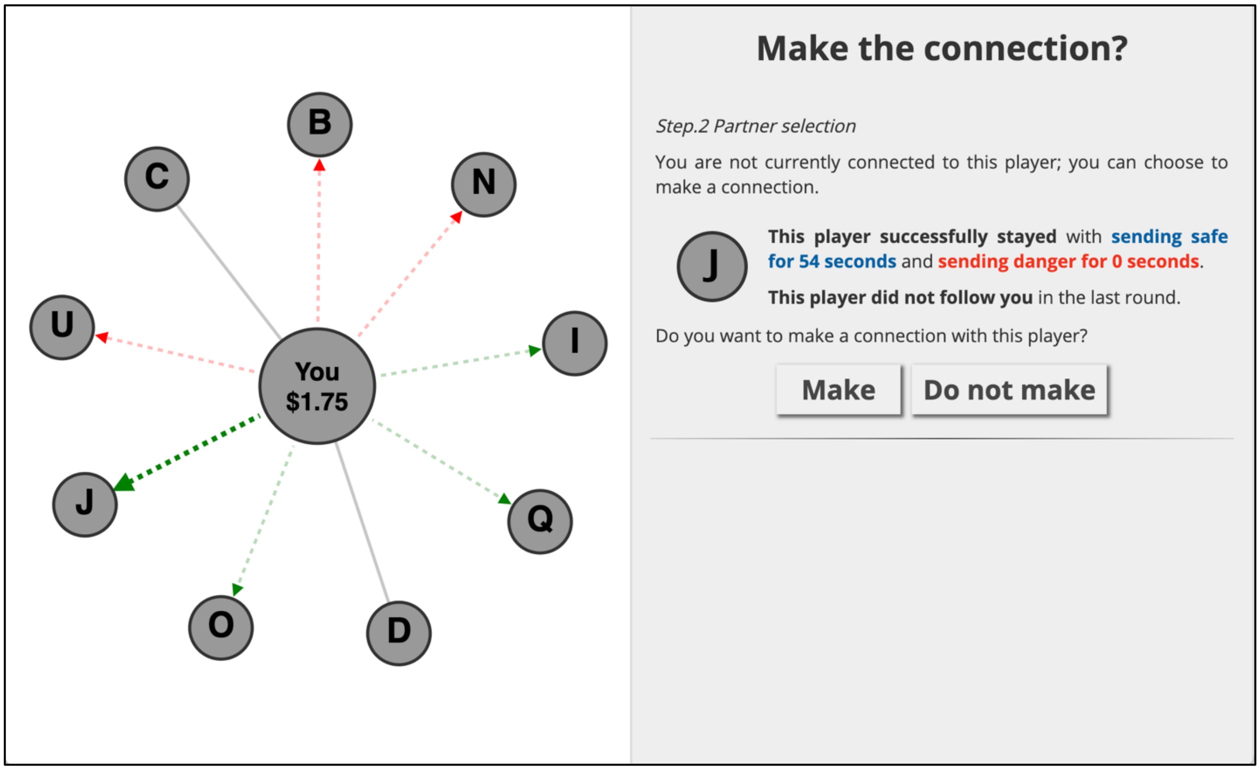
B
C
N
U
I
You
$1.75
J
Q
O
D
Make the connection?
Step.2 Partner selection
You are not currently connected to this player; you can choose to make a connection.
J
This player successfully stayed with sending safe for 54 seconds and sending danger for 0 seconds.
This player did not follow you in the last round.
Do you want to make a connection with this player?
Make
Do not make

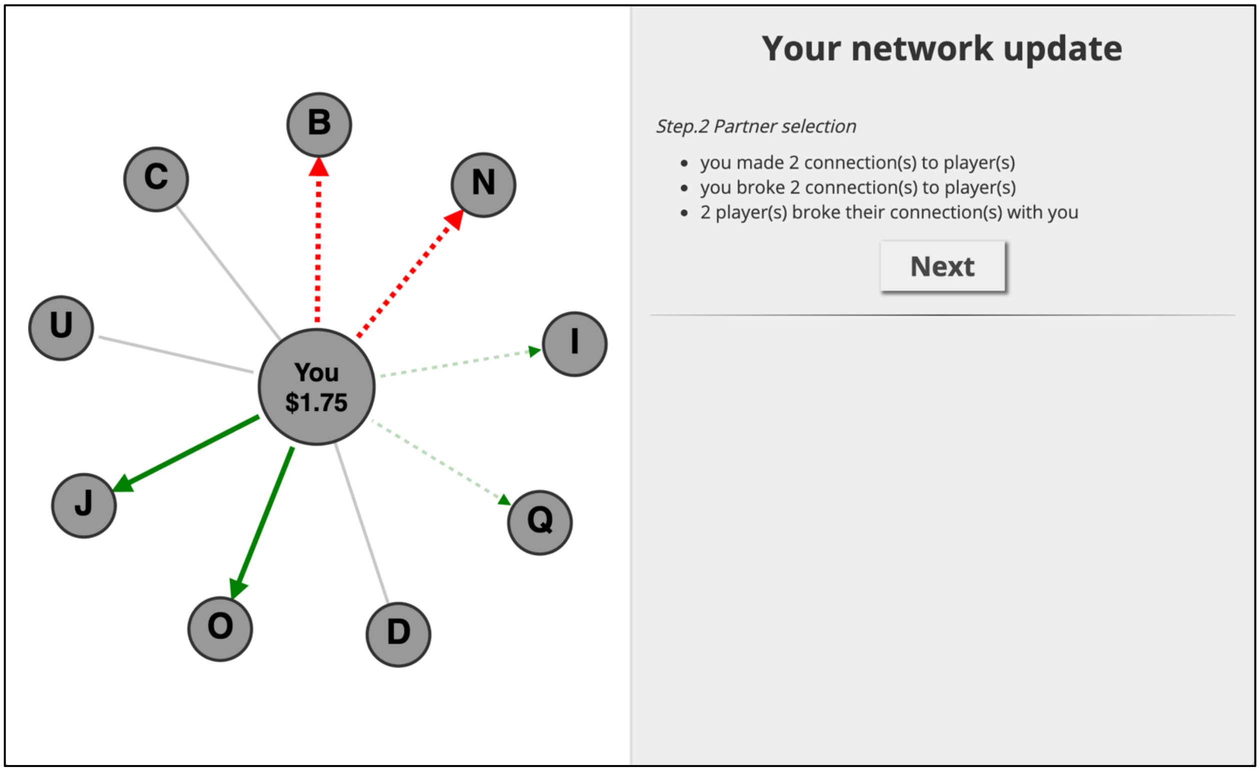
B
C
N
U
I
You
$1.75
J
Q
O
D
Your network update
Step.2 Partner selection
you made 2 connection(s) to player(s)
you broke 2 connection(s) to player(s)
2 player(s) broke their connection(s) with you
Next

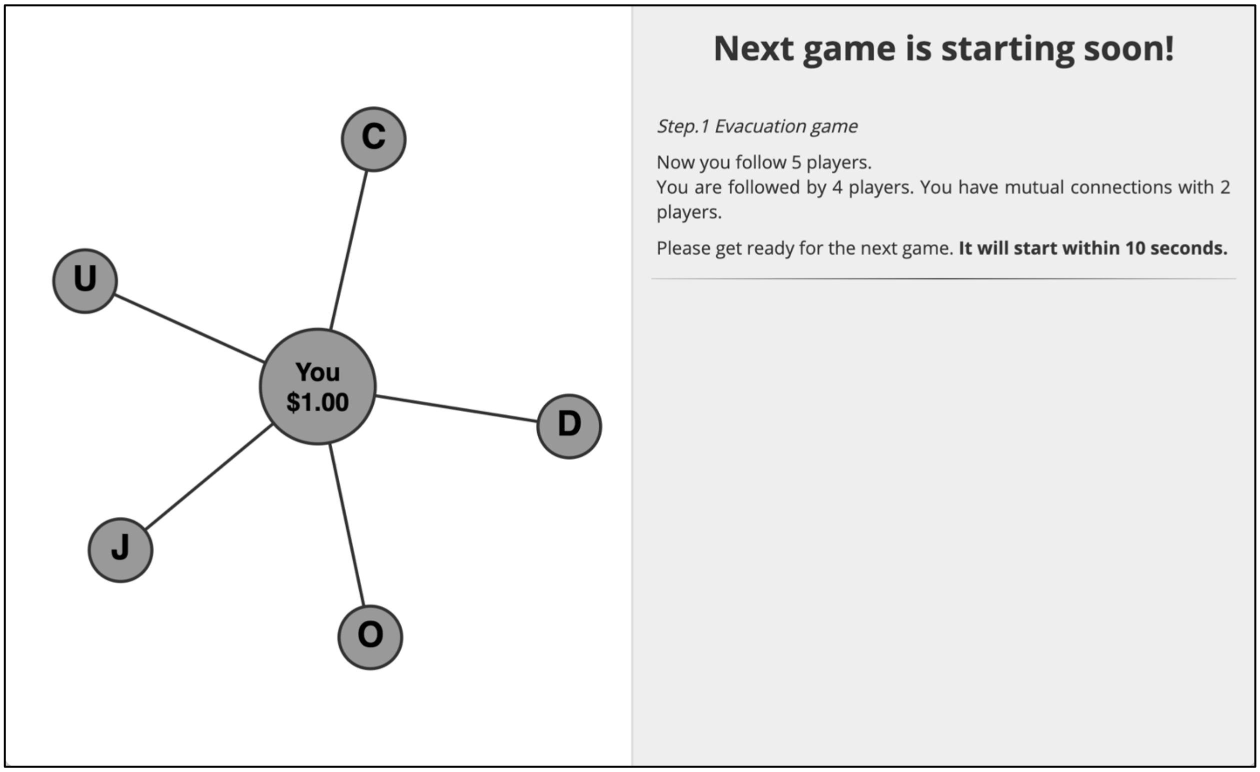
C
U
You
$1.00
D
J
O
Next game is starting soon!
Step.1 Evacuation game
Now you follow 5 players.
You are followed by 4 players. You have mutual connections with 2 players.
Please get ready for the next game. It will start within 10 seconds.

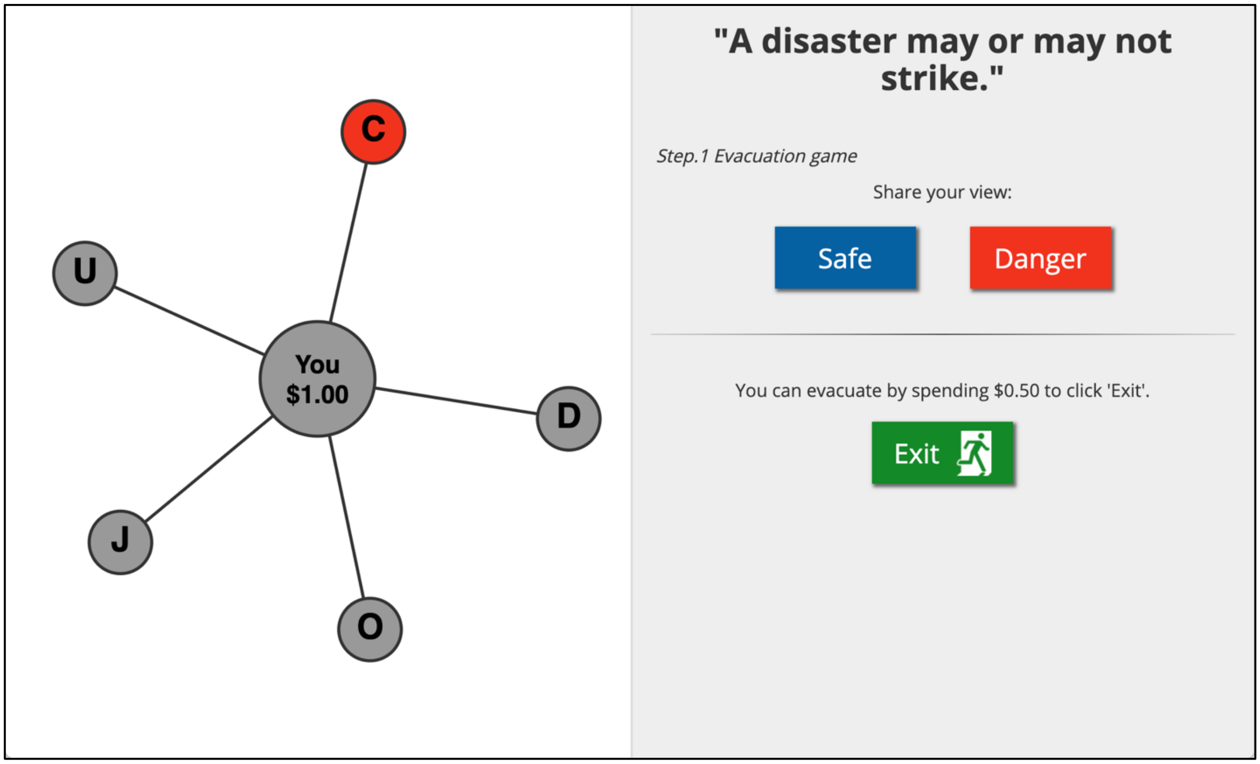
C
U
You
$1.00
D
J
O
"A disaster may or may not strike."
Step.1 Evacuation game
Share your view:
Safe
Danger
You can evacuate by spending $0.50 to click 'Exit'.
Exit

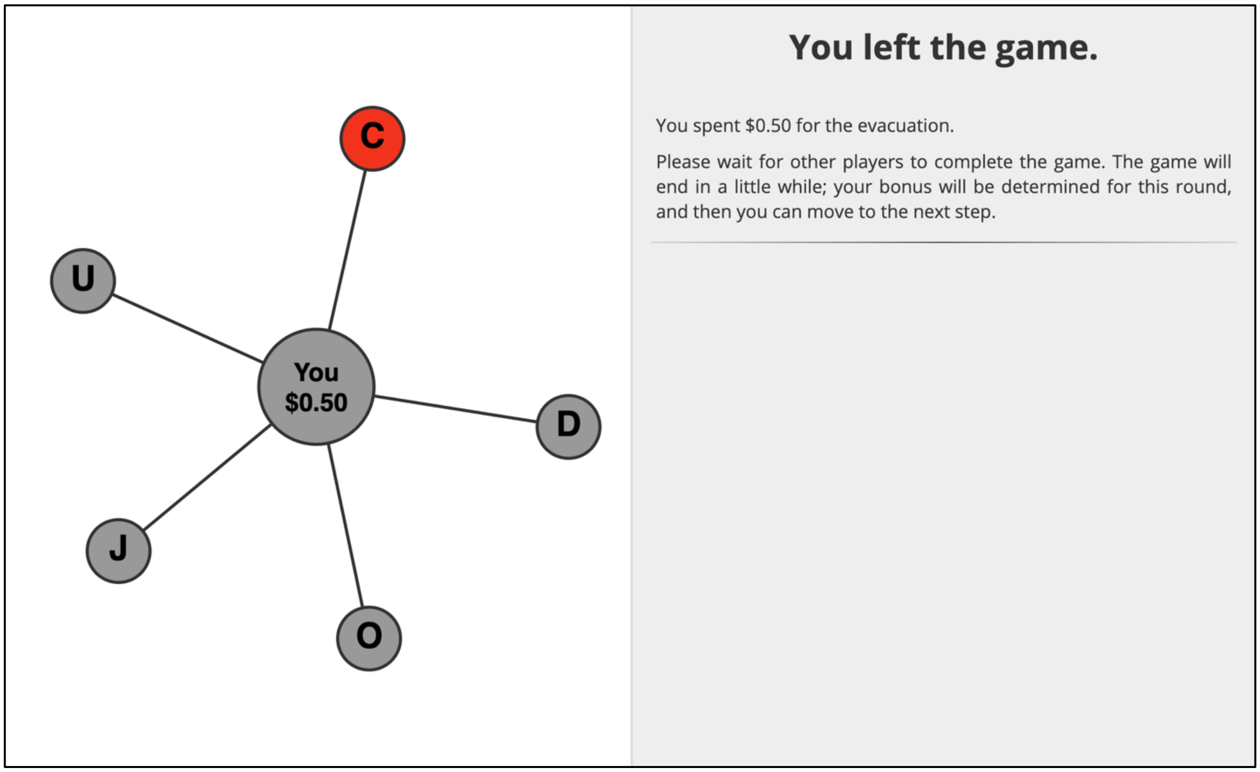
C
U
You
$0.50
D
J
O
You left the game.
You spent $0.50 for the evacuation.
Please wait for other players to complete the game. The game will end in a little while; your bonus will be determined for this round, and then you can move to the next step.

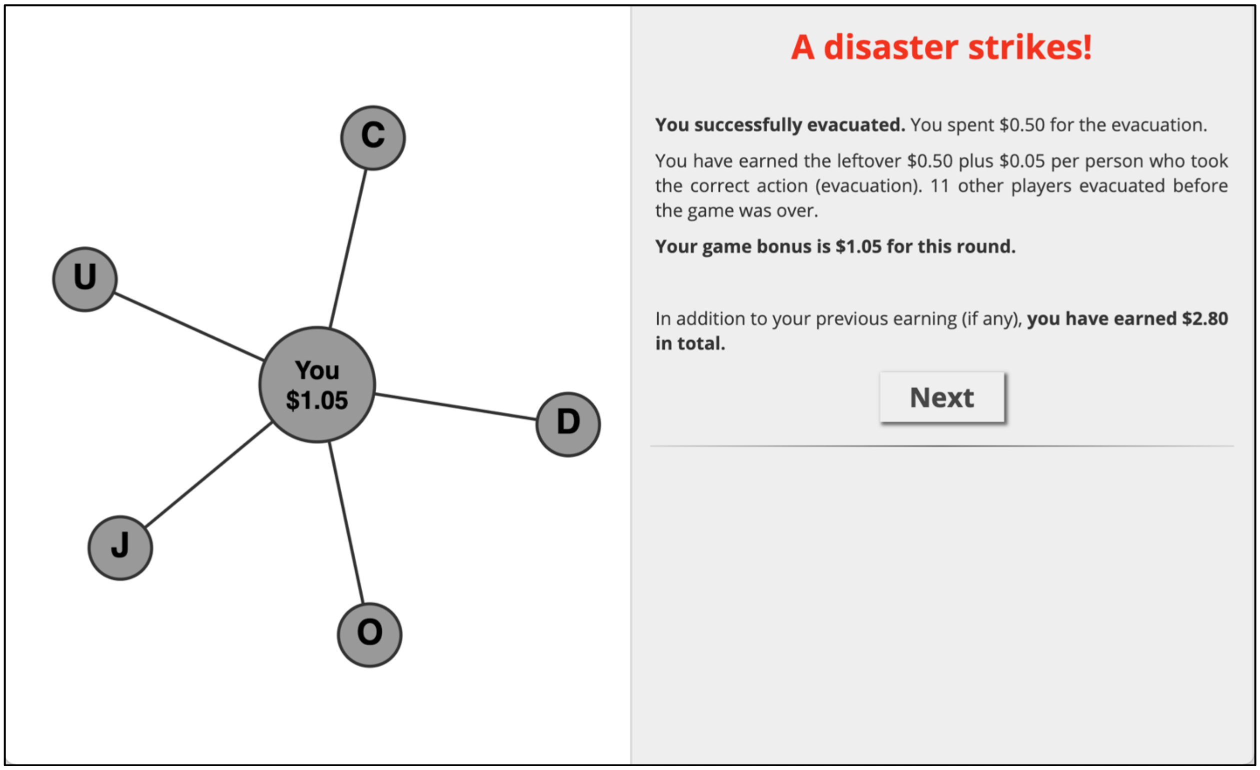
C
U
You
$1.05
D
J
O
A disaster strikes!
You successfully evacuated. You spent $0.50 for the evacuation.
You have earned the leftover $0.50 plus $0.05 per person who took the correct action (evacuation). 11 other players evacuated before the game was over.
Your game bonus is $1.05 for this round.
In addition to your previous earning (if any), you have earned $2.80 in total.
Next
